# Diabetes mellitus-driven pulmonary injury: multidimensional mechanisms linking metabolic dysregulation to gut-lung axis and promising therapies

**DOI:** 10.3389/fphar.2025.1689522

**Published:** 2025-10-23

**Authors:** Jiacheng Sun, Junyang Chen, Yuntian Shen, Xinlei Yao, Hualin Sun, Bingqian Chen, Jian Feng

**Affiliations:** ^1^ Department of Respiratory and Critical Care Medicine, Affiliated Hospital of Nantong University, Medical School of Nantong University, Nantong University, Nantong, Jiangsu, China; ^2^ Key Laboratory of Neuroregeneration of Jiangsu and Ministry of Education, Co-Innovation Center of Neuroregeneration, Nantong University, Nantong, Jiangsu, China; ^3^ Department of Orthopedics, Changshu Hospital Affiliated to Soochow University, First People’s Hospital of Changshu City, Changshu, Jiangsu, China

**Keywords:** diabetes mellitus, pulmonary injury, metabolic dysregulation, gut-lung axis, intestinal dysbiosis

## Abstract

Diabetes mellitus (DM), a globally prevalent metabolic disorder, poses a significant public health threat due to its systemic complications. Recent studies have increasingly recognized the lung as a target organ in diabetic pathology. However, owing to the respiratory system’s complex physiology, the mechanisms underlying DM-associated lung injury remain poorly understood and require further investigation. This review systematically elucidates the multifaceted effects of DM-induced metabolic disturbances on the lung, with a focus on four key pathophysiological axes triggered by hyperglycemic homeostasis, including chronic inflammation, oxidative stress imbalance, endocrine network disruption, and intestinal dysbiosis. Building upon the “metabolism-microbiota-immune” axis framework, this study demonstrates that: persistent hyperglycemia induces pulmonary tissue damage and immune microenvironment disruption through metabolite accumulation and mitochondrial dysfunction; DM-associated intestinal dysbiosis amplifies pulmonary inflammation via the gut-lung axis, mediated by metabolic reprogramming and immune cell trafficking; and metabolic aberration-driven dysregulation of innate/adaptive immunity serves as the pivotal mediator for progressive lung injury. Building on this mechanistic framework, we discuss emerging therapeutic avenues that target metabolic reprogramming, modulation of the gut microbiota, and restoration of immune homeostasis. Promising strategies include repurposed antidiabetic drugs (e.g., SGLT-2 inhibitors, GLP-1 receptor agonists), microbiome-targeted therapies (e.g., fecal microbiota transplantation), and novel immunomodulatory agents. These therapies are offering a new shift towards multi-target treatments for diabetic pulmonary complications.

## 1 Introduction

Diabetes mellitus (DM) is a systemic metabolic disorder characterized by chronic hyperglycemia resulting from insulin secretion defects and/or insulin resistance (Sacks et al., 2023). According to data from the International Diabetes Federation, the global prevalence of DM among adults aged 20–79 years reached 537 million in 2021, with projections indicating this number will rise to 693 million by 2045 (Cho et al., 2018). In recent years, advances in DM monitoring systems and in-depth research have led to significant progress in understanding the pathogenesis of DM-related complications. Among these, diabetic pulmonary injury has emerged as a novel target organ damage and is increasingly becoming a research focus (Lange et al., 1989; Ahmad et al., 2011). The clinical significance of respiratory complications in DM cannot be overlooked. Growing clinical evidence indicates that these complications pose substantial risks, with diabetic patients exhibiting a two-fold increase in pneumonia-associated mortality (Singh et al., 2020). Furthermore, chronic airway diseases affect approximately 20% of elderly diabetic individuals (Caughey et al., 2010). Moreover, a bidirectional association exists between DM and chronic obstructive pulmonary disease (COPD), with concomitant presentation of these conditions substantially complicating clinical management (Caughey et al., 2013). Collectively, these findings underscore the significant clinical implications of respiratory complications in diabetic patients.

At the pathological level, DM exerts multifaceted effects on pulmonary function through multiple mechanisms, including inflammatory responses, altered immune regulation, and pulmonary microvascular dysfunction (Tiengo et al., 2008; Erener, 2020; Roberts et al., 2018). Early experimental evidence from animal studies in 1997 demonstrated that DM combined with hyperlipidemia induces structural abnormalities in lung tissue, with these alterations being particularly pronounced in hyperlipidemia-associated DM (Popov and Simionescu, 1997). The metabolic disturbances of hyperglycemia, dyslipidemia, and insulin resistance in DM significantly alter systemic metabolic homeostasis. These pathological metabolic states play a crucial role in the development of diabetic complications (Eid et al., 2019). The lungs, with their extensive vascular network, are particularly vulnerable to circulating metabolic byproducts. This anatomical susceptibility suggests that diabetic pulmonary injury may be mechanistically linked to systemic metabolic dysregulation. Emerging evidence highlights the lung as a metabolically active organ, with recent studies identifying dysregulated metabolic pathways that contribute to both pulmonary injury and repair processes in lung diseases (Liu and Summer 2019; Rajesh et al., 2023). Evidence suggests that hyperglycemia directly impairs the metabolic function of pulmonary dendritic cells (DCs) (Nobs et al., 2023) and promotes systemic oxidative stress along with a pro-inflammatory state (Betteridge, 2000). Collectively, these findings indicate that the dysregulated metabolic milieu damages lung tissue through multiple mechanisms, including immune dysregulation, oxidative stress induction, and chronic inflammation.

Notably, the intestinal flora serves as a crucial metabolic regulatory hub, and its dysbiosis may exacerbate pulmonary immune imbalance through the gut-lung axis. Clinical evidence has revealed altered gut microbiome composition in diabetic patients (de Vos et al., 2022). Furthermore, intestinal dysbiosis may independently contribute to metabolic dysregulation. A growing body of evidence indicates that endocrine-disrupting chemicals, particularly those migrating from plastic products and food packaging additives, can induce gut microbiota dysbiosis through disruption of microbial homeostasis. This metabolic perturbation in intestinal flora has been mechanistically linked to impaired glucose regulation in mammalian hosts (Velmurugan et al., 2017). The pulmonary and intestinal mucosal immune systems exhibit bidirectional crosstalk, and DM-induced intestinal flora alterations may further compromise pulmonary mucosal barrier function (Yang et al., 2021; Özçam and Lynch, 2024). Collectively, these findings reveal that aberrant energy metabolism, dysregulated cell death, intestinal dysbiosis, and immune dysfunction collectively constitute the complex pathogenic network underlying diabetic pulmonary injury.

**FIGURE 1:**
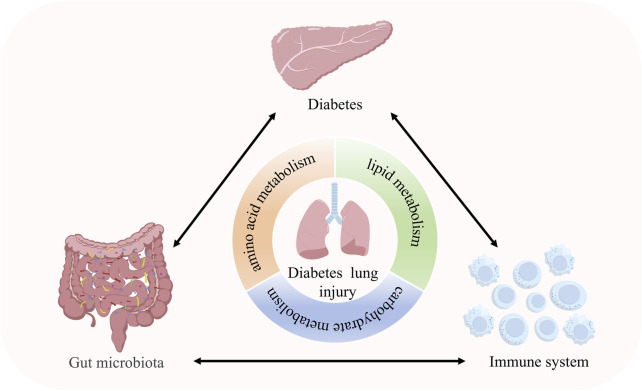
Crosstalk between metabolism, immunity and the gut microbiota in diabetic lung injury. Diabetic lung injury arises from the synergistic interplay between metabolic dysregulation, gut microbiota alterations, and immune dysfunction. This cooperative axis drives pulmonary pathology through gut-lung signaling, ultimately leading to endothelial barrier disruption, oxidative stress, and fibrotic remodeling.

Conventional DM research has predominantly focused on microvascular complications and neural damage, while the pathological mechanisms involving the lung—a highly metabolically active organ—have long been overlooked. Critical questions remain unresolved: How does metabolic dysregulation drive pulmonary injury through inter-organ crosstalk networks? What roles do the microbiome and immune system play in this process? As depicted in Figure 1, we posit that diabetic pulmonary injury results from a synergistic triad of metabolic dysregulation, gut microbiota alterations, and pulmonary immune defects, forming a self-sustaining vicious cycle. Here, we systematically evaluate this “gut-metabolism-immune” axis and highlight promising pharmacological strategies targeting its critical nodes.

## 2 DM-associated metabolic dysregulation

Diabetic pulmonary injury is conceptualized within a framework of a self-perpetuating vicious cycle, driven by the interplay of metabolic dysregulation, gut microbiota disruption, and immune imbalance. The analysis in this review thus begins by delineating the systemic metabolic disturbances inherent to diabetes. This section systematically examines the core mechanistic alterations in carbohydrate, lipid, and amino acid metabolism, which collectively establish the pathological foundation for the subsequent development of multi-organ complications.

In diabetic patients, impaired insulin secretion or insulin resistance disrupts metabolism and cell signal transduction (Sacks et al., 2023; Shahwa et al., 2022). Accumulation of advanced glycation end products (AGEs) resulting from aberrant glycation reactions plays a pivotal role in both DM progression and its associated complications (Khalid et al., 2022). The receptor for advanced glycation end products (RAGE), a transmembrane receptor for AGEs, is abundantly expressed in pulmonary tissue with highly selective localization to the basement membrane of type I alveolar epithelial cells (Fehrenbach et al., 1998). The RAGE pathway amplifies pulmonary damage induced by infection, direct injury, and inflammatory responses (Guo et al., 2012). Thus, the DM-AGEs-RAGE axis may represent a crucial mediator of diabetic pulmonary injury. Furthermore, in gestational diabetes mellitus (GDM) patients, pathological manifestations correlate with circulating AGEs levels (Li and Yang, 2019). Clinical evidence further links GDM to pulmonary sequelae in offspring: maternal hyperglycemia impairs fetal lung development (He et al., 2023) and is associated with reduced pulmonary function in progeny (Yang et al., 2024). Moreover, maternal-neonatal gut microbiota modifications and associated metabolic reprogramming can significantly impair neonatal pulmonary immune function (Stevens et al., 2022; Fonseca et al., 2021). These findings collectively reveal that maternal metabolic disturbances in DM can remotely impact fetal lung development across the placental barrier, closely associated with gut microbiota metabolism. These findings collectively reveal complex crosstalk among diabetic metabotypes, intestinal flora, and immune regulation.

Diabetic patients also showed characteristic lipid metabolism disorders (Golay et al., 1984). For example, disordered ketone body metabolism in DM impairs the synthesis and function of pulmonary surfactant (Jung et al., 2023). Thus, diabetic lipid metabolic alterations frequently elevate the risk of DM-related lung complications (Conte et al., 2024; Eid et al., 2023). In addition, lipid metabolism is closely related to the intestinal flora, which plays a crucial role in diabetic complications through its metabolites. And we will reveal this phenomenon below. This suggests that therapeutic modulation of microbiota shows clinical potential for mitigating diabetic complications.

Furthermore, amino acid metabolism is significantly dysregulated in patients with DM. Clinical evidence indicates that children with type 1 DM (T1DM) exhibit reduced levels of branched-chain amino acids (BCAAs) and decreased Fischer ratios (BCAA/AAA (aromatic amino acid)) (Hassan et al., 2023). Elevated levels of BCAAs (leucine, isoleucine, and valine) have been consistently observed in patients with type 2 DM (T2DM), potentially linking to both immune dysregulation and insulin resistance (Wang et al., 2011). Intriguingly, reduction of fasting plasma BCAAs levels alone is insufficient to improve insulin sensitivity, suggesting a potentially synergistic role of multiple tissues in modulating BCAAs metabolism to alter insulin responsiveness (Blair et al., 2023). The gut microbiota, however, serves as the master regulator of this metabolic network, where microbial BCAAs biosynthesis and catabolism dynamically control systemic BCAAs availability. Collectively, amino acid metabolic disorders can directly or indirectly affect lung function, and intestinal flora is the core regulator of this process.

## 3 Metabolic dysregulation: molecular bridges between DM and pulmonary injury

**FIGURE 2:**
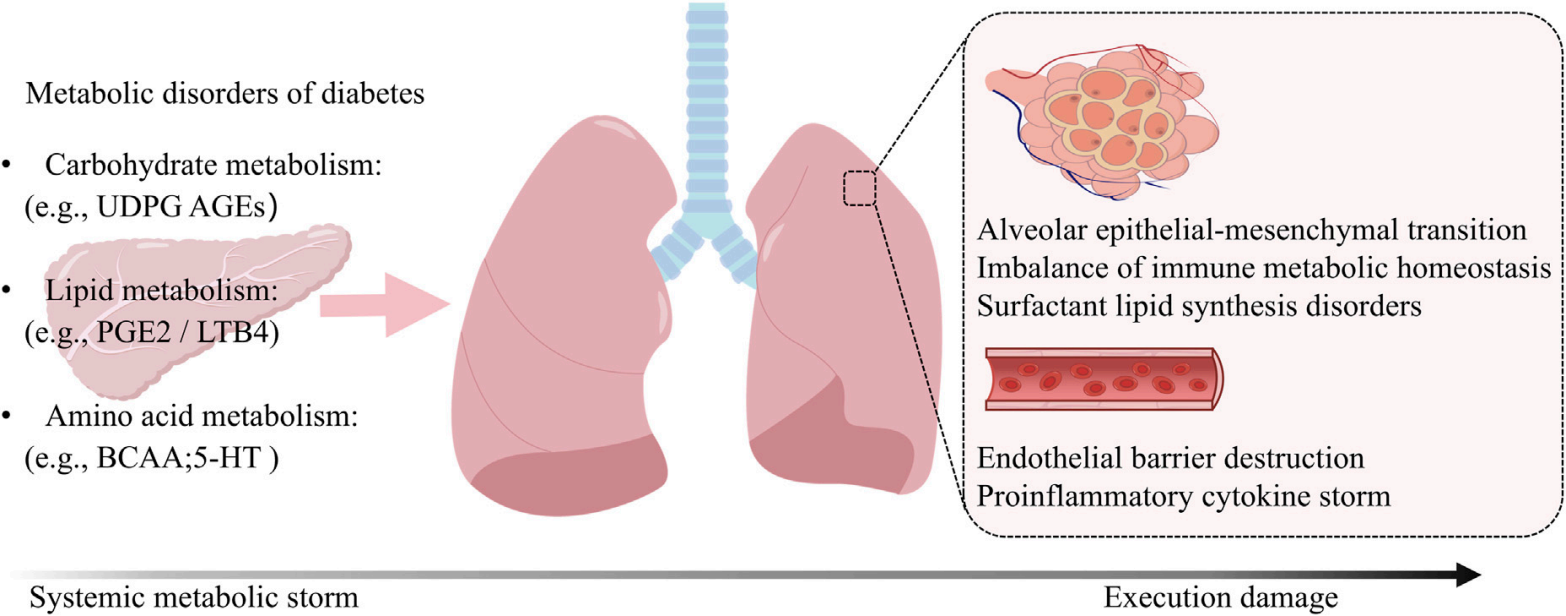
Metabolic Dysregulation as a Bridge from Diabetes to Lung Injury. Diabetes-induced imbalances in lipids, amino acids, and glucose generate pathogenic intermediates that disseminate systemically to the lung. This metabolic insult triggers pulmonary damage *via* key pathways: endothelial barrier breakdown, impaired tissue repair, inflammatory activation, and immune-metabolic reprogramming, collectively promoting fibrosis and infection susceptibility.

DM creates a pathological environment for multi-organ injury through comprehensive dysregulation of carbohydrate, lipid, and amino acid metabolism. Metabolic dysregulation serves as a molecular bridge linking diabetes to pulmonary injury, where systemic imbalances in lipids, amino acids, and glucose generate pathogenic intermediates that travel to the lungs, triggering endothelial barrier breakdown, impaired repair, inflammation, and immune metabolic alterations (Figure 2). Critically, hyperglycemia-mediated injury exhibits distinct organ-specific patterns: experimental animal models reveal tissue-specific metabolic reprogramming (Sas et al., 2016). Notably, metabolites such as AGEs, dysregulated ketone bodies, and BCAAs have been demonstrated to be directly or indirectly associated with structural abnormalities and functional decline in the lung (e.g., impaired surfactant synthesis, diminished respiratory muscle function). This establishes the metabolic basis for considering the lung a target organ in diabetes. Next, we will explore how these circulating metabolites disrupt intrapulmonary metabolic homeostasis, thereby directly linking systemic metabolic dysregulation to local pulmonary pathology.

Within the framework of DM-induced metabolic dysregulation (encompassing lipid/amino acid/carbohydrate metabolism), gut microbiota disruption, and loss of immune homeostasis, the lung emerges as a critical nexus of these systemic interactions. Although primarily regarded as a gas-exchange organ, the lung demonstrates remarkable metabolic activity, continuously enduring high oxidative stress and substantial circulatory demands. Its unique microenvironment—where an extensive capillary network interfaces with both inhaled pathogens and circulating metabolites—renders the pulmonary system particularly vulnerable to DM-induced metabolic perturbations.

### 3.1 Mechanisms of pulmonary metabolic homeostasis

Research indicates that pulmonary secretion, clearance, and other functions are sustained through metabolic pathways including glucose metabolism, lipid metabolism, and oxidative metabolism (Fisher, 1984). Key features of pulmonary glucose metabolism encompass glycolysis and the pentose phosphate pathway, which generate lactate, carbon dioxide, and intermediates for the biosynthesis of lipids, nucleic acids, and amino acids, along with biologically reduced NADPH, NADH and the high-energy compound ATP. Notably, a major fraction (40%–50%) of glucose is metabolized into lactate and pyruvate (Fisher, 1984), which subsequently contribute to fatty acid synthesis and incorporation into pulmonary surfactant lipids (Burgy et al., 2022). The synthesis of pulmonary surfactant represents a hallmark metabolic activity of the lung, which typically maintains a highly active state of lipid metabolism (Burgy et al., 2022). To ensure surfactant renewal, the lung utilizes circulating fatty acids from the bloodstream, which undergo intracellular remodeling before being incorporated into surfactant synthesis and secretion (Harayama et al., 2014). Emerging evidence indicates that pulmonary surfactant not only facilitates respiration but also contributes to host defense by promoting pathogen clearance and immune modulation (Han and Mallampalli, 2015). Furthermore, the lung enhances the clearance of circulating 5-hydroxytryptamine (5-HT; serotonin), a process potentially linked to the metabolic activity of pulmonary capillary endothelial cells and the platelet activation and recruitment (Yu et al., 2009). The serotonin transporter in pulmonary endothelial cells has been identified as a key regulator in these processes, participating in vascular remodeling and adaptation during neonatal transition (Castro et al., 2017). Collectively, these interconnected metabolic networks maintain pulmonary homeostasis, and their dysregulation may trigger pathological cascades.

Growing evidence has linked pulmonary diseases such as pulmonary hypertension, pulmonary fibrosis, and lung cancer to metabolic dysregulation (Rajesh et al., 2023; Xu et al., 2021; Koukourakis et al., 2017). The cellular diversity of the lung creates a complex microenvironment. Metabolites can relay signals through transcytosis across pulmonary endothelial cells (Jones and Minshall, 2020). For instance, mast cell-derived serotonin acts on 5-HT_2A_ receptors, enhancing methacholine-induced airway hyper-responsiveness in house dust mite-triggered experimental asthma (Mendez-Enriquez et al., 2021). Moreover, serotonin metabolites (5-hydroxyindoleacetic acid) derived from activated platelets and mast cells in the lung can act as ligands to drive eosinophil recruitment *via* the chemotactic receptor G protein-coupled receptor 35, exacerbating fungal infections (De Giovanni et al., 2023). Additionally, dysregulated lipid metabolism is a critical contributing factor in pulmonary diseases. In pulmonary fibrosis, alveolar type II epithelial cells generate foam cells and release them into the distal lung space in response to injury (Romero et al., 2015). Oxidized phospholipids subsequently accumulate in alveolar macrophages, enhancing the production of TGF-β1 and thereby exacerbating fibrotic progression (Romero et al., 2015). Moreover, elevated lactate metabolism is observed in fibrotic lung tissues, which induces pH-dependent activation of TGF-β, promoting fibroblast differentiation and driving fibrotic development (Kottmann et al., 2012). These findings demonstrate that dysregulated lipid metabolism serves as a metabolic reprogramming hallmark of pulmonary fibrosis. In summary, proper pulmonary metabolic function is crucial for maintaining lung homeostasis, and targeting aberrant metabolic pathways represents a promising therapeutic strategy for pulmonary disorders.

### 3.2 Energy metabolic dysregulation and diabetic pulmonary injury

The stable tricarboxylic acid (TCA) cycle sustains pulmonary surfactant synthesis through ATP production. However, the dynamic pulmonary microenvironment requires adaptive energy regulation *via* molecular sensors such as adenosine 5′-monophosphate (AMP)-activated protein kinase (AMPK) and the mammalian target of rapamycin (mTOR). AMPK activation serves to conserve cellular energy, whereas the mTOR pathway governs energy expenditure behaviors (Stapleton et al., 1996; Brown et al., 1994). In patients with DM, dysregulated AMPK coupled with mTORC1 activation drives lipid metabolic disorders and disease complications (Ruderman et al., 2013; Li et al., 2021; Uno et al., 2015). The mTOR signaling plays a pivotal role in pulmonary vascular remodeling, promoting pathological restructuring of pulmonary arterioles (Goncharova, 2013). Therapeutic targeting of both AMPK and mTOR may ameliorate lipid metabolism dysregulation, hyperglycemia, and DM-associated complications (Entezari et al., 2022; Rohm et al., 2022). Activation of the AMPK signaling pathway by metformin treatment in lipopolysaccharide (LPS)-induced acute pulmonary injury attenuates inflammatory responses (Zhang Y. et al., 2022). Similarly, AMPK activation in diabetic pulmonary injury models suppresses high glucose-driven alveolar epithelial-mesenchymal transition, revealing its dual role in maintaining energy homeostasis and preventing fibrosis (Wang L. et al., 2023). In summary, the AMPK/mTOR pathway plays a central role in maintaining pulmonary homeostasis by orchestrating energy metabolic balance. Its dysregulation is closely linked to DM-associated pulmonary injury, including fibrosis and vascular remodeling. Therapeutic strategies targeting this pathway—such as AMPK activation *via* metformin—demonstrate dual potential in ameliorating metabolic disturbances and suppressing pathological progression.

### 3.3 Lipid metabolism reprogramming: immune homeostasis disruption

DM sustains systemic chronic inflammation through lipid-mediated pathways, in which prostaglandins (PGs), particularly the abundant PGE2, serve as both dual mediators and amplifiers of inflammatory cascades. PGE2, an arachidonic acid (AA)-derived eicosanoid subclass, acts as a ubiquitous inflammatory mediator that can be potently suppressed by lipid emulsion therapy to mitigate tissue inflammation (Huang et al., 2020). In patients with DM, inflammatory cells markedly elevate the release of lipid mediator PGE2 in affected tissues, which serves as both a cause and consequence of inflammation (Martín-Vázquez et al., 2023). Concurrently, the LTB4 pathway is activated in DM, which upregulating ACE2/TMPRSS2 and arachidonate 5-lipoxygenase expression to exacerbate COVID-19 progression (Bonyek-Silva et al., 2021). Consistent with these findings, patients with COPD exhibit selective elevation of both PGE2 and LTB4 levels in exhaled breath condensate (Montuschi et al., 2003). These shared proinflammatory lipid signatures point to common pathogenic mechanisms driving multiorgan dysfunction in metabolic-pulmonary comorbidities, offering a mechanistic framework for understanding the shared pathophysiology between DM and COPD (Caughey et al., 2013; Hughes et al., 2020). Notably, elevated levels of 12(S)-hydroxyeicosatetraenoic acid [12(S)-HETE], a subclass of eicosanoids, activate the intracellular cation channel transient receptor potential vanilloid one and mediate endothelial dysfunction in DM (Otto et al., 2020). However, high concentrations of 12-HETE disrupt pulmonary immunity and compromise the pulmonary vascular endothelial barrier (Li et al., 2024; Zhang et al., 2018; Pernet et al., 2023). The DM-associated metabolic-immune axis further manifests in alveolar pathophysiology: obesity and T2DM induce alterations in surfactant synthesis within alveolar type II epithelial cells (Schipke et al., 2021). Notably, surfactant protein A (SP-A) and surfactant protein D (SP-D) serve dual roles as pulmonary innate immune proteins. Patients with DM exhibit reduced circulating SP-D levels that correlate with insulin resistance, whereas serum SP-A concentrations are elevated but show no significant association with systemic inflammation (Fernández-Real et al., 2010; Fernández-Real et al., 2008). Importantly, insulin signaling has been demonstrated to be essential for post-inflammatory SP-D recovery (Fernández-Real et al., 2010). These findings underscore metabolic regulation as a critical determinant of pulmonary immunometabolic homeostasis.

### 3.4 Abnormal amino acid metabolism: BCAA and tryptophan are double-edged swords

As outlined above, BCAA metabolism is severely dysregulated in DM. Systemic BCAA imbalance exerts direct effects on pulmonary function. The β-aminoisobutyric acid, a catabolic derivative of BCAAs, activates the AMPK/Nrf2 pathway to attenuate pulmonary ischemia-reperfusion injury (Zhang et al., 2023). In DM, impaired BCAAs catabolism may disrupt pulmonary repair mechanisms, potentially exacerbating post-injury complications (Lynch and Adams, 2014). Notably, the diaphragm exhibits robust BCAAs metabolism (Huang et al., 2011). Clinical investigations have established that decreased Fischer ratios are significantly associated with both diminished respiratory muscle performance and declined pulmonary function (Yoneda et al., 1992). Consequently, BCAAs metabolic disturbances in T1DM patients may represent a pathophysiological mechanism underlying the observed pulmonary functional alterations. The clinical correlation between DM and lung cancer remains contentious, with population studies demonstrating discordant survival outcomes (Khateeb et al., 2019; Shieh et al., 2012; Hatlen et al., 2011). Mechanistically, non-small cell lung cancer (NSCLC) cells exhibit enhanced BCAAs uptake (Mayers et al., 2016). The elevated BCAAs catabolism in NSCLC cells suppresses m6A demethylase ALKBH5 expression, thereby promoting epithelial-mesenchymal transition and tumor metastasis (Mao et al., 2024). However, increased plasma BCAAs levels have been observed in some NSCLC patients (Wang Y. et al., 2021), potentially attributable to BCAAs catabolic inhibition by branched-chain keto acid dehydrogenase kinase in NSCLC (Xue et al., 2023). These findings demonstrate that BCAAs metabolism is reprogrammed to support tumor growth in the context of lung cancer. This metabolic plasticity likely underlies the observed clinical heterogeneity, as DM-induced systemic metabolic disturbances may interact with tumor-specific BCAAs flux to modulate disease progression.

Tryptophan metabolic dysregulation plays a pivotal role in DM pathogenesis through three primary pathways, including the indole, kynurenine, and serotonin pathways (Gao et al., 2023). Clinical evidence demonstrates that patients with DM exhibit significantly elevated kynurenine levels and increased kynurenine/tryptophan ratios (Kozieł and Urbanska, 2023), which are strongly associated with exacerbated pulmonary inflammatory responses during COVID-19 infection (Conte et al., 2024; Thomas et al., 2020). Moreover, 5-hydroxytryptamine (5-HT) serves as a potent vasoactive mediator of tissue edema and inflammation. 5-HT induces airway plasma extravasation through transient receptor potential vanilloid 4 (TRPV4) (Retamal et al., 2021), a cation channel critical for epithelial and endothelial barrier integrity. Under diabetic and hypertensive conditions, aberrant TRPV4 signaling disrupts pulmonary epithelial/endothelial homeostasis, establishing a vicious cycle of edema and inflammation (Darby et al., 2016). Therapeutic targeting of this pivotal cation channel emerges as a promising strategy for DM-associated pulmonary complications, underscoring its translational potential.

## 4 Molecular mechanisms of pulmonary injury in DM

**FIGURE 3:**
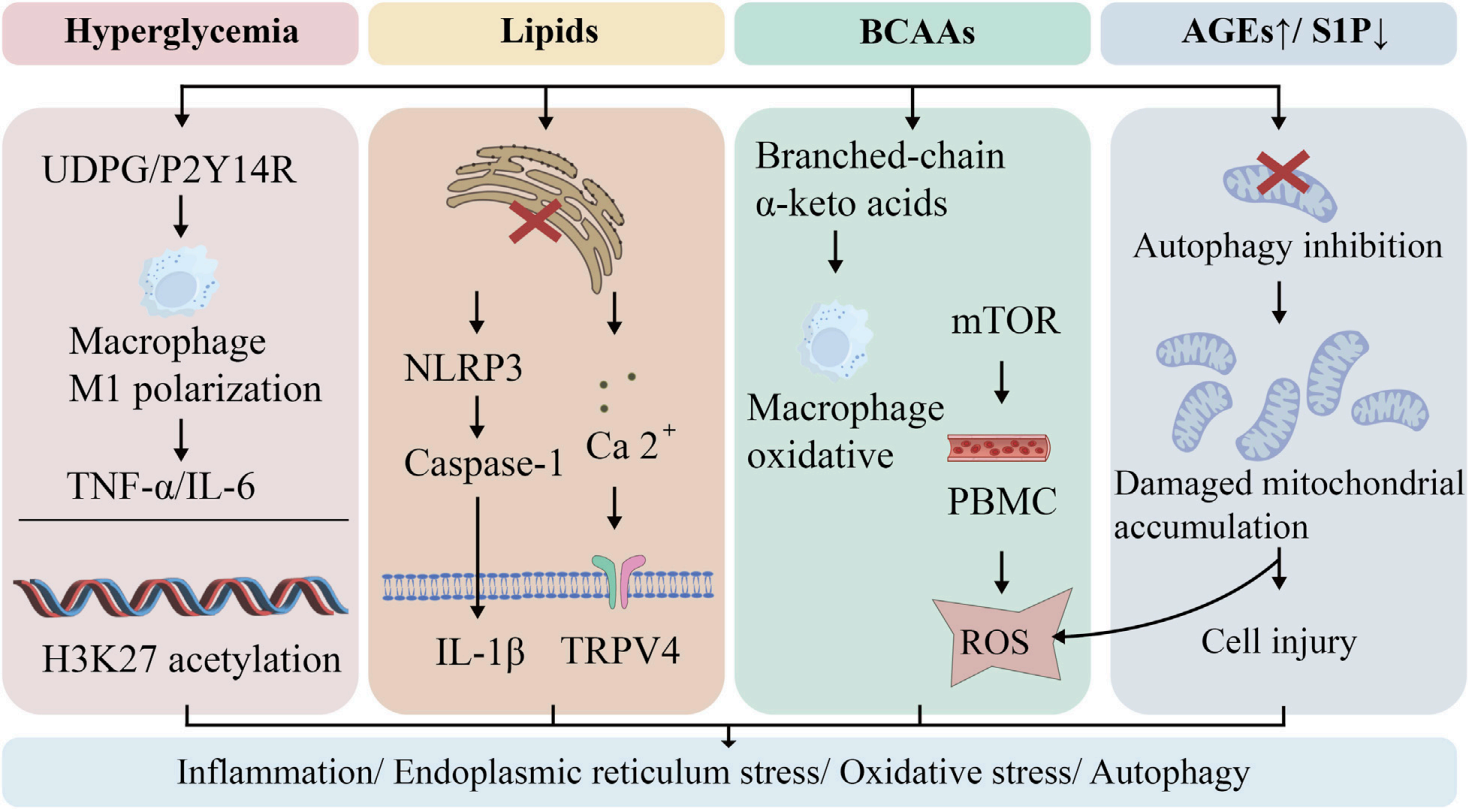
Core Mechanistic Network in Diabetic Pulmonary Injury. Diabetic conditions trigger synchronized immunometabolic disturbances in the lung through multiple signaling pathways. Hyperglycemia disrupts glycogen metabolism, leading to UDPG accumulation and P2Y14R-mediated pro-inflammatory macrophage polarization. Concurrently, elevated BCAAs activate mTOR signaling and generate toxic metabolites, while lipotoxicity and hyperglycemia induce ERS and NLRP3 inflammasome activation. These pathways converge to promote oxidative stress, impaired mitochondrial clearance, and epigenetic modifications, collectively establishing a persistent inflammatory microenvironment that drives progressive lung damage.

Having established that metabolic dysregulation disrupts pulmonary homeostasis, we now examine how these insults propagate into distinct molecular damage signals. This section delineates the core cellular stress pathways—including endoplasmic reticulum stress, oxidative stress, mitophagy dysfunction, and epigenetic reprogramming—that drive pulmonary injury in diabetes at the molecular level (Figure 3).

### 4.1 Inflammation-driven pulmonary injury

Circulating inflammatory cells are recruited and secrete inflammatory mediators, which communicate with structural cells (including epithelial cells, endothelial cells and fibroblasts) to trigger tissue inflammation and injury. DM is characterized by disordered glucose metabolism, manifested as decreased glycogen synthesis and increased glycogenolysis. Evidence indicates that loss of UDP-glucose pyrophosphorylase two reduces intracellular glycogen levels and impairs N-glycosylation targets (Wolfe et al., 2021). Notably, we detected upregulated glycogen and N-linked glycans in the fibrotic cores of lung tissue sections from pulmonary fibrosis patients, with lysosomes contributing to glycogen utilization during fibrogenesis (Conroy et al., 2023). Studies show that glycogen metabolism upregulates uridine diphosphate glucose (UDPG) in macrophages (Ma et al., 2020). Binding to the P2Y14R receptor, UDPG synergistically drives macrophage polarization toward a proinflammatory phenotype—characterized by robust TNF-α/IL-6 secretion—via enhancing both STAT1 expression and phosphorylation (Figure 3) (Ma et al., 2020). The pivotal inflammatory role of this pathway is corroborated in P2Y14R-deficient mouse models (Ka et al., 2021). In diabetes, impaired skeletal muscle glycogen synthase activity may disrupt UDPG clearance (Majer et al., 1996), while glutamine metabolism, which possesses UDPG-lowering potential, is itself dysregulated (Petrus et al., 2020; Shi et al., 2024), collectively exacerbating inflammation mediated by the UDPG/P2Y14R axis. We therefore hypothesize that these aberrant glycogen metabolic pathways may collectively contribute to inflammation-driven pulmonary injury in DM.

### 4.2 ERS-driven pulmonary injury

The endoplasmic reticulum (ER) is a functionally versatile nutrient-sensing platform that plays a critical role in metabolic regulation. Endoplasmic reticulum stress (ERS) has been identified as a major contributor to human diseases, including DM, neurodegenerative disorders, and cancer (Hetz and Papa, 2018). Emerging evidence indicates that ERS disrupts lipid homeostasis and interferes with critical cell signaling pathways in pulmonary disorders, with its role in DM-associated pulmonary injury gaining increasing recognition (Hsu et al., 2017; Chen et al., 2020). ERS not only initiates inflammatory cascades but also determines cellular fate (Hetz and Papa, 2018). Mechanistic investigations reveal that ERS activates the cytosolic pattern recognition receptors NOD1 and NOD2 (nucleotide-binding oligomerization domain-containing proteins one and 2) through bacterial peptidoglycan detection (Zangara et al., 2021), subsequently inducing inflammation and IL-6-dominated cytokine production (Keestra-Gounder et al., 2016). Notably, as a crucial cytosolic pattern recognition receptor, the NLRP3 inflammasome detects diverse stimuli, including microbial pathogen-associated molecular patterns (PAMPs) and damage-associated molecular patterns (DAMPs), while also responding to metabolic disturbances such as hyperglycemia and free fatty acids (Zangara et al., 2021). These findings suggest that ERS-induced inflammatory responses may be mechanistically linked to NLRP3 inflammasome activation (Figure 3). In calcium signaling regulation, the ER serves as the primary intracellular calcium reservoir, with calcium release playing a pivotal role in signal transduction. Research has demonstrated that the DM-associated protein CD36 promotes substantial release of arachidonic acid and PGE2 by modulating ERS-dependent calcium influx, thereby triggering inflammatory cascades (Kuda et al., 2011). Moreover, administration of human islet amyloid polypeptide in diabetic mice was found to induce β-cell dysfunction, concomitant with activation of TRPV4 channels and subsequent ERS induction (Casas et al., 2008). Similar mechanisms were observed in the substantia nigra of Parkinson’s disease mouse models, where TRPV4 signaling was demonstrated to mediate both ERS and inflammatory pathways (Liu et al., 2022). Consistent with these findings, TRPV4 overexpression in human lung adenocarcinoma A549 cells elevated intracellular calcium concentrations and induced ERS (Yoo et al., 2020). Collectively, these results suggest that ERS, functioning as a metabolic stress response system, may transduce diabetic stress into pulmonary tissue damage through circulating mediators.

### 4.3 Oxidative stress-driven pulmonary injury

Oxidative stress represents a core pathogenic driver in the development and progression of DM. Under pathological conditions including obesity, insulin resistance, hyperglycemia, chronic inflammation and dyslipidemia, excessive reactive oxygen species (ROS) production occurs (Forrester et al., 2018). Notably, the pulmonary system inherently exists in a high redox-pressure environment, where ROS overaccumulation induces both DNA damage and lipid peroxidation - molecular mechanisms now established as central to DM-associated pulmonary injury (Thannickal and Fanburg, 2000). Emerging evidence highlights the pivotal role of BCAAs in modulating oxidative stress responses and their downstream effects (Figure 3). As previously noted, diabetic patients exhibit impaired BCAAs metabolism leading to systemic accumulation. Mechanistically, elevated BCAA slevels exacerbate oxidative stress through two distinct pathways: (i) increased circulating BCAAs enhance ROS production in peripheral blood mononuclear cells (PBMCs) *via* mTOR signaling hyperactivation, resulting in PBMC overactivation (Zhenyukh et al., 2017); (ii) defective BCAAs catabolism causes accumulation of branched-chain α-keto acids, which induce macrophage oxidative stress and perpetuate chronic inflammatory states (Liu S. et al., 2021). Furthermore, dysfunctional brown adipose tissue (BAT) in diabetic patients contributes to this pathogenic cascade (Okla et al., 2017). Specifically, impaired mitochondrial BCAAs flux and reduced synthesis of BCAA-derived metabolites in BAT exacerbate hepatic oxidative stress while depleting glutathione reserves (Verkerke et al., 2024). This ‘dual-’hit’ mechanism - combining enhanced oxidative stress with compromised redox regulation - not only establishes a pathological link between diabetic metabolic dysregulation and tissue damage, but also suggests the therapeutic potential of modulating BCAAs metabolism to restore redox homeostasis.

### 4.4 Mitophagy-driven pulmonary injury

In the pathological progression of DM, aberrant accumulation of AGEs disrupts mitophagic homeostasis, thereby exacerbating multi-organ damage (Dewanjee et al., 2022; Xu R. et al., 2023; Zhao et al., 2024). Notably, pulmonary tissue repair processes demonstrate particular dependence on proper autophagic regulation and glucose metabolic function (Li et al., 2019), a characteristic that may fundamentally explain the enhanced pulmonary vulnerability observed in diabetic patients. From the perspective of lipid metabolism, reduced levels of sphingosine-1-phosphate (S1P) in diabetic mouse models lead to significant sphingolipid metabolic defects, which impair both autophagic function and T-cell activity (Jin et al., 2024). More critically, this mechanism is also observed in human lung microvascular endothelial cells, where impaired S1P/S1PR1 signaling directly compromises autophagy, contributing to pulmonary vascular damage (Goel et al., 2021). Collectively, these findings indicate that DM-associated metabolic dysregulation can directly induce pulmonary vascular injury by disrupting autophagy. Notably, autophagy dysfunction and metabolic disturbances form a vicious cycle - impaired autophagic flux exacerbates glucose and lipid metabolic imbalances (Kim and Lee, 2014), which may serve as a key driver for the progressive metabolic deterioration in DM (Figure 3). This recognition provides novel mechanistic insights into the pathogenesis of multi-organ complications in diabetic patients.

### 4.5 Epigenetic dysregulation-driven pulmonary injury

Previous study has demonstrated complex genetic risk variants during DM pathogenesis (Gao et al., 2019). These variants not only determine disease susceptibility but also reshape tissue-specific epigenomic landscapes (Ling et al., 2022). In T1DM, for instance, genetic variations at risk loci are strongly associated with characteristic inflammatory responses and distinct patterns of immune dysregulation (Gao et al., 2019; Chiou et al., 2021). Persistent hyperglycemia and lipotoxicity synergistically exacerbate metabolic dysregulation through epigenetic modifications such as histone H3 lysine 27 acetylation (H3K27ac). Mechanistic studies reveal that H3K27ac accelerates diabetic cardiomyopathy progression by activating the long non-coding RNA PPARα-seRNA (Ma et al., 2024). Notably, H3K27ac-mediated chromatin remodeling exhibits cell-type-specific effects in pulmonary tissues: in alveolar fibroblasts, it transcriptionally represses autophagy *via* LINC00941 activation (Zhang J. et al., 2022), whereas in macrophages, serum- and glucocorticoid-inducible kinase 1 (SGK1) disrupts immune homeostasis through H3K27ac-dependent reprogramming (Wu et al., 2024). These findings collectively demonstrate that hyperglycemia-induced chromatin remodeling persistently influences fibroblast activation. Consequently, targeted modulation of dynamic epigenetic marks like H3K27ac may emerge as a novel therapeutic strategy for mitigating metabolic complications and enabling precision risk prediction. Current evidence strongly establishes metabolic dysregulation as a pivotal factor in the pathogenesis of DM and its complications. However, elucidating the precise mechanisms through which diabetic metabolic disorders induce pulmonary tissue injury remains a significant challenge. While abnormal accumulation of metabolic intermediates has been shown to drive pulmonary inflammation and injury (Conroy et al., 2023; Kwon et al., 2019), the mechanistic underpinnings for most metabolites remain undefined, necessitating further investigation to delineate the molecular pathways of metabolite-driven lung damage in DM. Building upon existing findings, glycogen metabolism emerges as a potential therapeutic target, with glycogen synthase modulators and receptor for RAGE antagonists demonstrating promising therapeutic potential.

## 5 Gut microbiota in DM: metabolic crosstalk with pulmonary pathology

The mechanisms discussed thus far have largely focused on the direct pathogenic effects of metabolic dysregulation in the lung. However, the full picture of diabetic pulmonary injury necessitates a systemic perspective that incorporates the crucial role of the gut microbiota. Diabetes-driven gut dysbiosis acts as a key amplifier, exacerbating systemic metabolic and immune perturbations. The following sections will elucidate how the gut microbiota and its metabolites mediate remote control of pulmonary pathology *via* the gut-lung axis.

**FIGURE 4:**
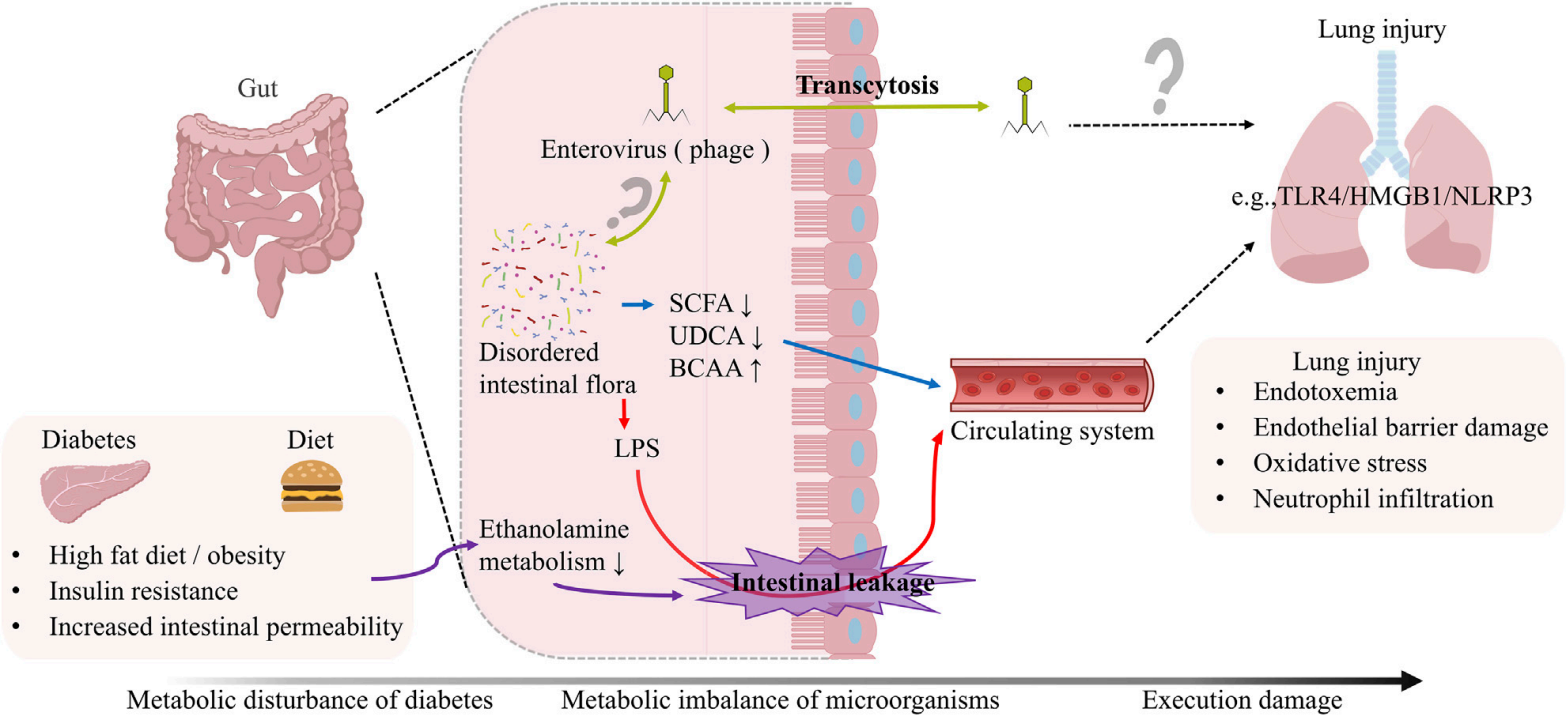
The Gut-Lung Axis in Diabetic Pulmonary Pathogenesis. The gut-lung axis mediates diabetic pulmonary injury through circulating microbial metabolites and bacterial components. Diabetes-induced gut dysbiosis results in reduced SCFA-producing bacteria, impaired bile acid metabolism, and increased gut permeability. This permits translocation of LPS, BCAAs, and other microbial products into systemic circulation. Upon reaching the lung, these gut-derived mediators activate innate immune receptors, triggering endothelial activation, neutrophilic infiltration, and oxidative stress responses that collectively drive pulmonary inflammation and tissue remodeling.

Current evidence has firmly established that intestinal dysbiosis represents a critical pathological component in the development and progression of DM (Yang et al., 2021). The mechanistic interplay between the gut microbiome and host chronic inflammatory states/metabolic disturbances in diabetic patients has garnered increasing research attention (Chávez-Talavera et al., 2017). The small intestine serves as the primary site for lipid reabsorption, where gut microbiota-derived metabolites modulate immune homeostasis (Greer et al., 2013). Emerging evidence suggests that targeted modulation of gut microbial composition and its metabolic output may confer beneficial effects against DM-associated complications (Canfora et al., 2019). Similarly, in T2DM, altered microbial metabolism of histidine leads to elevated imidazole propionate levels (Molinaro et al., 2020), which subsequently exacerbates glucose metabolic dysregulation. Emerging evidence demonstrates significant suppression of ethanolamine-metabolizing microbiota in the gut of individuals with DM and obesity, resulting in ethanolamine accumulation. This metabolic perturbation significantly downregulates the expression of intestinal tight junction proteins, particularly ZO-1 (Mishra et al., 2023), thereby initiating a multistep pathogenic cascade: (i) compromised intestinal barrier integrity promotes bacterial translocation and systemic dissemination of deleterious metabolites; (ii) subsequent activation of systemic inflammatory responses; and (iii) consequent aggravation of insulin resistance, deterioration of glucose homeostasis, and disruption of immunological equilibrium - ultimately precipitating multi-organ dysfunction. As illustrated in Figure 4, the gut microbiota serves as a central hub in diabetic lung injury, where gut-derived metabolites and toxins (e.g., LPS, BCAAs) remotely regulate pulmonary inflammation and immune responses through systemic circulation, ultimately leading to endothelial disruption and oxidative stress.

### 5.1 Metabolite-mediated intestinal-pulmonary axis crosstalk

Gut microbial metabolites, serving as pivotal mediators of host-microbiota interactions, exhibit dynamic composition and functionality regulated by host genetics, dietary patterns, and environmental factors, while reciprocally modulating host homeostasis *via* the metabolism-immune axis (Schoeler and Caesar, 2019). These bioactive molecules, like short-chain fatty acids (SCFAs) and BCAAs, can enter systemic circulation by crossing the intestinal barrier, thereby modulating the function of distal organs such as the lungs *via* metabolite-organ crosstalk. During the pathological progression of DM, four classes of critical microbial metabolites (SCFAs, BCAAs, bile acids, bacterial endotoxins) mediate multi-organ damage through the gut-lung axis (Saad et al., 2016). In conclusion, gut microbial metabolites, regulated by host genetics/diet/environment, mediate DM multi-organ damage *via* gut-lung axis mechanisms.

SCFAs, the principal metabolic end-products of dietary fiber fermentation by obligate anaerobes (e.g., *Bacteroides* spp.), play a pivotal role in maintaining intestinal redox homeostasis (van Hoek and Merks, 2012). Mechanistic investigations demonstrate that SCFAs markedly enhance insulin sensitivity and optimize energy metabolism in diabetic patients through G protein-coupled receptor activation (Gao et al., 2009). Notably, pulmonary tissues lack essential substrates for SCFA biosynthesis (e.g., dietary fiber), and germ-free mouse models exhibit significantly reduced pulmonary SCFA levels - direct evidence that lung SCFAs are entirely dependent on functional gut microbiota-derived interorgan supply (Liu Q. et al., 2021). In conclusion, SCFAs from gut microbiota enhance insulin sensitivity, maintain intestinal redox, and supply lungs *via* interorgan transport.

BCAAs (comprising leucine, isoleucine, and valine), as essential amino acids, exhibit metabolic dynamics tightly regulated by the gut microbiota—which participates in both BCAAs synthesis (*Prevotella* spp.) and catabolism (*Roseburia* spp.) (Gojda and Cahova, 2021). Mechanistic studies reveal that high-fat diets elevate circulating BCAAs levels, activating the mTORC1 signaling pathway in skeletal muscle, thereby inducing lipid deposition and ultimately promoting obesity-associated insulin resistance (Newgard et al., 2009). Based on the above analysis, gut microbiota-regulated BCAAs drive mTORC1-mediated muscle lipid accumulation and insulin resistance in high-fat diet-induced obesity.

Notably, bile acids form a dynamic regulatory network through enterohepatic circulation. Primary bile acids (e.g., cholic acid) are converted into secondary bile acids (e.g., deoxycholic acid) *via* microbial 7α-dehydroxylation, which activate FXR and TGR5 receptors to not only improve glucose and lipid metabolism in diabetic patients but also exert potent anti-inflammatory effects (Chávez-Talavera et al., 2017). Of particular significance, ursodeoxycholic acid (UDCA), as a key secondary bile acid, exhibits immunomodulatory functions through a dual mechanism: downregulating CD86 expression on DCs while simultaneously inhibiting Th2 cell differentiation and promoting Treg cell proliferation, thereby effectively ameliorating eosinophilic airway inflammation (Willart et al., 2012). In conclusion, microbial-converted bile acids activate FXR/TGR5 to improve metabolism and inflammation, while UDCA modulates DCs and Tregs to ameliorate eosinophilic airway inflammation.

The pathological association between DM and increased intestinal permeability coupled with gut microbiota dysbiosis has garnered substantial scientific attention. Substantial evidence confirms that gut barrier dysfunction-induced microbial imbalance facilitates bacterial endotoxin (e.g., LPS) translocation across the intestinal epithelium into systemic circulation, precipitating metabolic endotoxemia - a process driving multi-tissue injury through systemic inflammatory responses (Saad et al., 2016). Notably, LPS-CD14 binding *via* pattern recognition receptors not only mediates innate immune activation but has been mechanistically linked to insulin resistance, obesity, and DM pathogenesis (Cani et al., 2007). To sum up, gut barrier dysfunction in DM drives microbiota-LPS translocation, triggering CD14-mediated systemic inflammation and insulin resistance, exacerbating multi-organ damage.

From a systems biology perspective, gut microbial metabolites serve as critical signaling mediators in gut-lung axis crosstalk, whose homeostatic disruption may reprogram metabolic-immune networks to propagate diabetic multi-organ complications. This trans-organ pathophysiological interconnection provides a novel conceptual framework for understanding DM-associated systemic damage.

### 5.2 Bacterial endotoxins in DM-associated pulmonary injury

Mounting experimental evidence continues to delineate the crucial involvement of gut-derived endotoxins in pulmonary injury pathogenesis through pattern recognition receptor (PRR)-dependent molecular crosstalk. Mechanistically, microbiota-derived endotoxins orchestrate parallel activation of both caspase-11-mediated non-canonical inflammasome pathways and canonical caspase-1/NLRP3 inflammasome complexes. This dual activation cascade induces catastrophic breakdown of alveolar-capillary barrier integrity, manifesting pathologically as alveolar edema, neutrophilic inflammation, and refractory hypoxemia (Cheng et al., 2017; Jin et al., 2021). In conclusion, gut endotoxins trigger lung injury *via* PRR-dependent dual inflammasome pathways, disrupting alveolar-capillary integrity.

The Toll-like receptor 4 (TLR4) system emerges as the principal lipopolysaccharide (LPS) recognition apparatus, executing transmembrane signaling through myeloid differentiation primary response 88 (MyD88)-dependent phosphorylation cascades (Poltorak et al., 1998). Of particular pathogenic significance, intestinal epithelial TLR4 activation stimulates systemic release of high-mobility group box 1 (HMGB1), a prototypic damage-associated molecular pattern (DAMP) that mediates remote pulmonary parenchymal injury *via* hematogenous dissemination (Sodhi et al., 2015). This mechanistic paradigm is substantiated by the complete abrogation of pulmonary injury phenotypes in intestinal epithelium-specific TLR4 knockout murine models (Sodhi et al., 2015). Based on the above analysis, TLR4 recognizes LPS *via* MyD88, triggering gut HMGB1 release that causes lung injury through hematogenous spread.

This gut-lung interorgan communication mechanism demonstrates significant clinical relevance in chronic respiratory diseases, with large-scale cohort studies establishing a strong inverse correlation between gut microbiota α-diversity reduction and key clinical parameters including asthma exacerbation frequency and annual lung function decline in COPD (Tangedal et al., 2024). In traumatic shock models, gut barrier collapse generates proinflammatory mediators that form a “second hit” through mesenteric lymphatic circulation, exacerbating acute pulmonary injury and precipitating multi-organ dysfunction (Magnotti et al., 1998). Collectively, these findings demonstrate that gut-derived inflammatory cascades mediated by TLRs/NLRs signaling pathways serve not only as key drivers of experimental pulmonary injury, but their persistent activation likely contributes to the pathological progression of advanced diabetic complications (e.g., diabetic lung disease). This paradigm provides novel therapeutic targets for multi-organ protection in metabolic disorders.

### 5.3 Gut virome dynamics in DM progression

Emerging research has progressively unveiled the dynamic evolution of the gut virome during DM progression and its complications. Contemporary evidence reveals that specific bacteriophages (e.g., *Caudovirales* members) exert critical regulatory functions in glucolipid metabolic disorders by modulating host-bacterial interaction networks (Fan et al., 2023). Notably, the ecologically integrated system formed by the gut virome and bacteriome generates distinct microbial metabolite signatures across DM subtypes through niche-specific regulatory mechanisms governed by “phage predation-bacterial lysis” dynamics (Nishijima et al., 2022; Hu et al., 2023). These cross-kingdom virus-bacteria interactions exhibit systemic regulatory potential, as circulating bacteriophages can translocate across the intestinal barrier *via* epithelial transcytosis, contributing to immune homeostasis in distal organs such as pancreatic islets and adipose tissue (Nguyen et al., 2017). Clinical interventions suggest that nutritional modulation (e.g., dietary fiber supplementation) may attenuate DM-associated gut dysbiosis by recalibrating temperate-lytic phage cycle equilibria (Avellaneda-Franco et al., 2024). Nevertheless, population-level virome heterogeneity and its interplay with host genetic determinants demand systematic exploration. Emerging synthetic biology tools enabling precision phage editing may pioneer novel therapeutic paradigms for metabolic reprogramming through “top-down” microbial ecosystem engineering.

In environmental exposure research, a paradigm-shifting discovery has redefined pulmonary microbiology: the lung parenchyma, previously deemed sterile, hosts a unique microbial ecosystem shaped by microbial migration-colonization-clearance dynamics (Natalini et al., 2023). This breakthrough elucidates mechanisms whereby environmental pollutants exacerbate respiratory pathologies *via* microbiota-immune axis disruption. Significantly, pulmonary microbiome dysbiosis in DM may constitute a novel mediator of gut-lung axis crosstalk, expanding our understanding of systemic complications.

Regarding DM-associated gastrointestinal pathophysiology, studies demonstrate hyperglycemia-driven systemic inflammation through three key mechanisms: (1) Downregulation of intestinal tight junction proteins (e.g., ZO-1) enhances gut permeability, enabling systemic translocation of pathogen-associated molecular patterns (PAMPs, e.g., LPS); (2) Reduced microbiota β-diversity generates individualized metabolic phenotypes, with specific bacterial consortia (e.g., Bacteroidetes/Firmicutes ratios) exhibiting strong correlations with insulin sensitivity; and (3) Gut-derived outer membrane vesicles mediate multi-organ injury *via* lymphatic trafficking (Yang et al., 2021). While animal models have partially deciphered these mechanisms, human studies face substantial challenges: interindividual microbiota variability and confounding environmental exposures (e.g., antibiotic history) complicate causal inference. From a translational perspective, microbiome signature-guided interventions—including precision probiotics and engineered phage cocktails—show therapeutic promise, though their efficacy necessitates validation through multicenter randomized controlled trials.

## 6 Dysregulation of the pulmonary immune microenvironment

**FIGURE 5:**
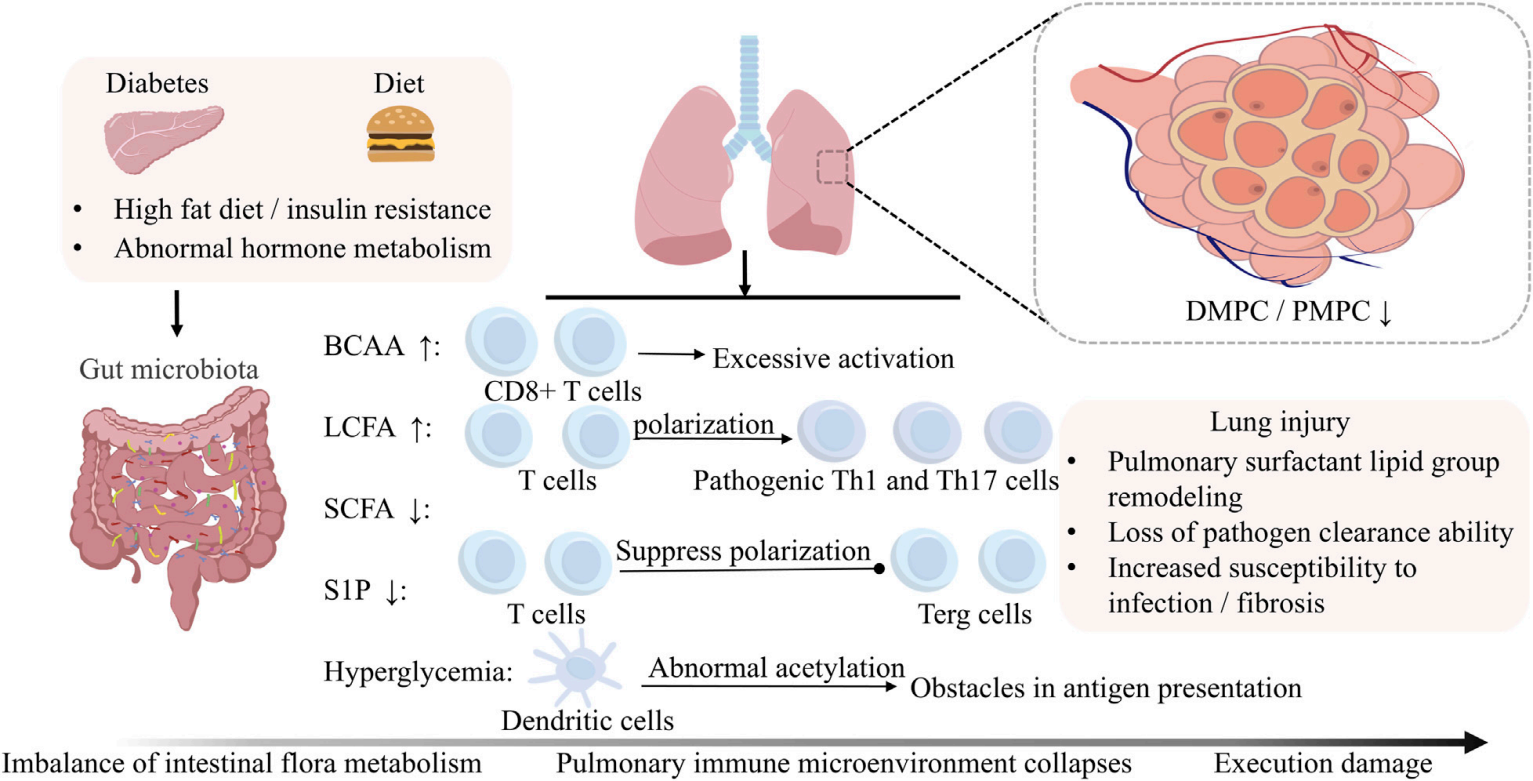
Microbiota-Driven Immune Dysregulation in the Diabetic Lung. Gut microbiota disturbances disrupt pulmonary immune homeostasis through metabolite-mediated mechanisms. First, metabolite imbalances disrupt adaptive immunity: SCFAs depletion impairs Treg differentiation, BCAAs accumulation hyperactivates CD8^+^ T cell glycolysis, and LCFAs excess promotes Th1/Th17 polarization. Concurrently, innate immunity collapses. This collapse involves S1P deficiency-mediated arrest of Treg autophagy, hyperglycemia-induced DC hyperacetylation, which disrupts antigen presentation, and DMPC/PMPC depletion that cripples surfactant-dependent pathogen clearance. Furthermore, adaptive immunity undergoes metabolic reprogramming; BCAA-PD-1 crosstalk alters the efficacy of NSCLC immunotherapy, and CD8^+^ memory T cells exhibit failure of the gluconeogenesis-glycogen axis.

Gut microbiota, through its metabolites and immunomodulatory functions, constitutes a critical hub linking diabetes to pulmonary pathologies. Signals originating from the gut and circulation ultimately converge within the pulmonary immune microenvironment, determining the balance between inflammation and immune tolerance. Next, we will elucidate how diabetes and its associated metabolic and microbiota dysregulation disrupt the equilibrium of innate and adaptive immune cells in the lung, leading to uncontrolled inflammation and tissue damage (Figure 5).

The pulmonary immune defense system operates through two interconnected arms: the innate immune network (encompassing neutrophils, macrophages, eosinophils, mast cells, natural killer cells, γδ T cells, innate lymphoid cells, and dendritic cells) and the adaptive immune apparatus (T and B lymphocytes). Under diabetic conditions, synergistic dysregulation of immune and metabolic homeostasis fosters a chronic inflammatory microenvironment, predisposing to pulmonary tissue injury. Established research confirms that diabetes mellitus (DM) and hyperlipidemia compromise pulmonary defense mechanisms by disrupting surfactant biosynthesis. Crucially, DM-associated metabolic dysregulation extends beyond biosynthetic disruption to critically impair surfactant’s antimicrobial functionality. In murine models of diet-induced obesity, pulmonary tissues and bronchoalveolar lavage fluid demonstrate significant depletion of critical phospholipids—dimyristoyl phosphatidylcholine (DMPC) and palmitoyl myristoyl phosphatidylcholine (PMPC). These dysregulated phospholipid profiles not only hinder pathogen clearance but also enhance SARS-CoV-2 infectivity by modulating viral entry mechanisms (Du et al., 2022). Therefore, diabetic metabolic dysregulation critically subverts pulmonary immune resilience by destabilizing surfactant integrity, amplifying infection susceptibility through combined biosynthetic, antimicrobial, and lipidome perturbations.

From a metabolic regulatory perspective, DM-induced hormonal dysregulation impairs pulmonary immunity through two principal pathways: First, hyperactivated glucocorticoid signaling suppresses alveolar macrophage phagocytic capacity; second, systemic insulin resistance reprograms immunometabolic circuits, driving pro-inflammatory polarization of immune cells (Webber et al., 2022). Emerging evidence further identifies gut microbiota dysbiosis in DM as a key driver of pulmonary immunopathology *via* systemic immune-metabolic axis activation. Gut-derived metabolites interact with pulmonary immune effectors, establishing a self-amplifying inflammatory loop within lung tissue (Burcelin, 2016). This gut-lung axis mechanism has been experimentally validated in COPD and COVID-19 pathobiology, where specific microbial taxa regulate pulmonary immune responses through Th17/Treg balance modulation and associated cytokine networks (Lai et al., 2022; Nagata et al., 2023). Therefore, DM orchestrates multi-organ immunometabolic crosstalk through endocrine-immune circuitry and gut-lung microbial networks, collectively reprogramming pulmonary defense landscapes toward chronic inflammation and pathogenic vulnerability.

Current findings underscore that maintaining metabolic homeostasis is paramount for pulmonary immune equilibrium, with gut microbiota serving as central metabolic regulators. Therapeutic modulation of microbiota-derived metabolites—particularly SCFAs and secondary bile acids—through dietary interventions or pharmacological agents shows significant promise in counteracting DM-associated pulmonary immune dysfunction, potentially *via* epigenetic modulation of immune cell function.

### 6.1 Gut microbiota and the immune microenvironment

Gut microbiota metabolism serves as a central immunoregulatory hub, coordinating immune system development and functional maturation through multifactorial mechanisms. During early-life immune programming, vertical microbial transmission from mother to offspring establishes a protective neonatal gut ecosystem. This process not only initiates primary microbial colonization but also activates TLR-dependent signaling to drive immune effector differentiation, establishing the foundation for lifelong immunological tolerance (Koren et al., 2024). Therefore, early-life gut microbiota-host interactions critically shape immunological trajectories by integrating microbial colonization, innate receptor signaling, and tolerance programming, forming an essential developmental axis for pulmonary and systemic immune homeostasis.

Gut microbiota-derived SCFAs are key messengers of the gut-lung axis (Figure 5). At the immune level, metabolic crosstalk critically shapes polarization: persistent long-chain fatty acid (LCFA) exposure depletes intestinal SCFAs while promoting differentiation of naïve T cells into pro-inflammatory Th1/Th17 subsets in the small intestine (Haghikia et al., 2015). In contrast, SCFA enrichment induces dual regulatory mechanisms—JNK1/p38 signaling suppression and Foxp3 transcriptional activation—to promote regulatory T (Treg) cell differentiation, thereby attenuating inflammatory cascades (Haghikia et al., 2015; Smith et al., 2013). Therefore, the metabolic milieu directs T cell lineage commitment through opposing lipid-sensing pathways, wherein SCFA-mediated immunomodulation counterbalances pro-inflammatory fatty acid signaling to maintain mucosal immune equilibrium and restrain pathological inflammation.

This metabolically driven immunomodulation exhibits trans-organ synergy across mucosal systems. The gut-lung axis facilitates dynamic interorgan communication, with gut-derived metabolites modulating distal immunity. Seminal work demonstrates that systemic BCG vaccination triggers intestinal lamina propria-resident innate memory cells *via* microbiota-dependent spatiotemporal metabolic reprogramming, identifying a paradigm for distal mucosal immune memory establishment (Jeyanathan et al., 2022). Therefore, this trans-mucosal metabolic dialogue establishes a systemic immune memory architecture, wherein gut-orchestrated metabolic reprogramming by microbial and vaccine stimuli epigenetically primes distal barrier sites for enhanced pathogen vigilance through conserved immuno-metabolic circuitry.

Emerging research has further elucidated that two major mucosal interfaces achieve systemic regulation through metabolite-immune crosstalk. On one hand, gut-to-lung axis: Circulating gut-derived metabolites (e.g., tryptophan catabolites) directly regulate pulmonary epithelial immunity; on the other hand, lung-to-gut feedback: Respiratory inflammatory signals reciprocally remodel gut microbial composition, forming a self-reinforcing metabolic-immune network (Özçam and Lynch, 2024). This trans-mucosal metabolic-immune synergy provides a novel theoretical framework for understanding microbiota-mediated systemic immune reprogramming.

### 6.2 Dysregulation of the innate immune system

T1DM is defined by immunopathology rooted in multifactorial regulatory T cell (Treg) dysfunction, with collapse of this central immune checkpoint driving persistent immune dysregulation (Kukreja et al., 2002). Tregs maintain immune homeostasis through two synergistic pathways: (1) paracrine release of immunosuppressive cytokines (e.g., IL-10, TGF-β) and (2) direct cellular interactions with innate immune populations—including monocyte-derived macrophages, granulocytes, dendritic cells (DCs), and innate lymphoid cells. Mechanistic drivers of Treg impairment converge on autophagy dysregulation. In diabetic murine models, sphingolipid metabolic disruption—marked by sphingosine-1-phosphate (S1P) deficiency—triggers mTOR-dependent autophagosome overproduction, accelerating Foxp3 proteasomal degradation and crippling Treg suppressive function (Jin et al., 2024). Therapeutic S1P restoration rescues Treg fitness *via* S1PR1-STAT5 signaling, which stabilizes Foxp3 expression and rebalances autophagic flux through lysosomal reactivation (Jin et al., 2024). Therefore, sphingolipid-autophagy crosstalk constitutes a pivotal regulatory nexus governing Treg stability, whose diabetic disruption propagates T1DM immunopathology by crippling immunosuppressive circuits, while pharmacologically restoring this axis emerges as a strategic therapeutic paradigm for re-establishing immune tolerance (Figure 5).

DCs exhibit parallel metabolism-driven dysfunction in T1DM, with subset-specific roles shaping immune polarization: cDC1 skews toward Th1 induction, while pDC preferentially promotes immune tolerance (Lo and Clare-Salzler, 2006). In diabetic pathology, DCs display metabolically programmed functional decay: Impaired efferocytosis in wound-resident DCs correlates with lysosomal acidification defects and dysregulated LC3-II expression (Masch et al., 2022). SLC7A11 inhibition restores efferocytic capacity by rescuing cystine/glutamate antiporter-dependent lysosomal proteolysis, accelerating diabetic wound repair (Masch et al., 2022). Hyperglycemia-induced epigenetic reprogramming in pulmonary DCs suppresses MHC-II and costimulatory molecule expression *via* histone hyperacetylation, blunting antigen presentation and CD8^+^ T-cell priming (Figure 5). HDAC inhibitors reverse these defects, reinstating DC immunogenicity (Nobs et al., 2023). Therapeutic implications: T1DM-associated metabolic insults—S1P depletion, glucolipotoxicity—converge on autophagic and epigenetic mechanisms to disable immunoregulatory cells. Targeting sphingolipid metabolism (e.g., S1P analogs), glucose transporters, or HDACs may restore tissue immunometabolic equilibrium, offering novel intervention strategies.

### 6.3 Pathological remodeling of adaptive immunity

The adaptive immune system orchestrates pathogen defense and tumor immunosurveillance through metabolic reprogramming, with CD8^+^ T cell remodeling serving as a linchpin. Mechanistic studies identify a tripartite metabolic axis—gluconeogenesis, glycogen metabolism, and the pentose phosphate pathway (PPP)—as essential for memory T cell survival. Cytosolic phosphoenolpyruvate carboxykinase (PCK1) catalyzes oxaloacetate-to-glucose-6-phosphate conversion, diverting metabolic flux toward glycogen synthesis and PPP activation. The resultant NADPH sustains Tm longevity by preserving glutathione redox homeostasis (Ma et al., 2018). This metabolic shunt enables Tm cells to bypass glycolysis-dependent energy production, maintaining functionality in nutrient-poor niches.

In addition to their direct effects on lung tissue, BCAAs also serve as critical immune-related metabolites (Figure 5). At physiological levels, BCAAs enhance CD8^+^ T cell antitumor activity *via* FoxO1-mediated glucose transporter 1 (GLUT1) upregulation, facilitating glycolytic-oxidative phosphorylation coupling (Yao et al., 2023). Clinically, gut microbiota-derived BCAAs synergize with PD-1 inhibitors to improve therapeutic responses in non-small cell lung cancer (NSCLC), potentially through microbiota-driven expansion of peripheral Tm cells and natural killer (NK) cell activation (Yao et al., 2023; Zhu et al., 2023; Jin et al., 2019). Strikingly, BCAAs metabolism displays paradoxical roles in diabetic pathophysiology—gut dysbiosis-induced BCAA accumulation confers survival benefits in specific NSCLC-DM cohorts, yet effect directionality depends on individual metabotypes, necessitating longitudinal metabolomics to decode the “DM-lung cancer” risk continuum (Qian et al., 2022). NSCLC patients exhibit aberrant expression of DM-specific microbial dysbiosis markers (CRP, LBP, CD14), implicating microbiota-derived endotoxemia in lung carcinogenesis (Qian et al., 2022). Therefore, BCAA metabolism manifests as a pleiotropic immunological arbitrator, where gut microbiota-driven metabolic reprogramming and host metabotype specificity converge to dictate its paradoxical roles across cancer immunotherapy and diabetic comorbidity, mandating precision metabologenomic approaches to harness its therapeutic duality while mitigating DM-associated oncogenic risk.

Integrating these findings, we propose a tripartite metabolic-immune axis driving DM-associated pulmonary injury. Firstly, pulmonary surfactant lipidome remodeling: DM-induced sphingolipid dysregulation (e.g., sphingosine-1-phosphate depletion) and phospholipid restructuring (reduced DMPC/PMPC ratios) compromise alveolar barrier integrity. Secondly, microbiota metabolite-driven immunometabolic reprogramming: Microbial metabolites (BCAAs, SCFAs) epigenetically modulate immunoregulatory cells (Tregs, DCs) *via* histone hyperacetylation and JNK1/p38-Foxp3 signaling. Finally, endocrine-immune integration: The gut-lung axis mediates systemic crosstalk through a microbiota-hormone-immunity network, where hyperglycemia-induced metabolic memory persistently impairs DC antigen presentation *via* HDAC-dependent epigenetic silencing.

Future investigations should prioritize resolving three pivotal scientific questions: (1) Whether microbial antigens drive lung tissue-specific autoimmune responses *via* molecular mimicry or related mechanisms; (2) How distinct metabolites (e.g., S1P vs BCAAs); cooperatively regulate immune cell fate determination across spatiotemporal dynamics; (3) Strategies to re-establish pulmonary immunometabolic homeostasis through microbiota-targeted metabolic interventions. Therapeutic strategies addressing these mechanisms—such as S1PR agonists or microbiota transplantation combined with immune checkpoint inhibitors—may pioneer a precision therapeutic paradigm for DM-associated pulmonary complications.

## 7 Therapeutic strategies for DM-associated pulmonary injury

The escalating global prevalence of DM necessitates future advancements in novel biomarker discovery and personalized therapeutic strategies. Clinically, managing pulmonary complications in DM confronts five cardinal challenges: the intricate molecular crosstalk governing metabolic-immune networks; the translational gaps in preclinical animal models recapitulating human-specific immunometabolic pathologies; the absence of standardized efficacy metrics for tissue-specific immunomodulation; the polypharmacy challenges arising from multimorbidity management; and the undercharacterized risks of off-target drug-metabolite interactions.

**FIGURE 6:**
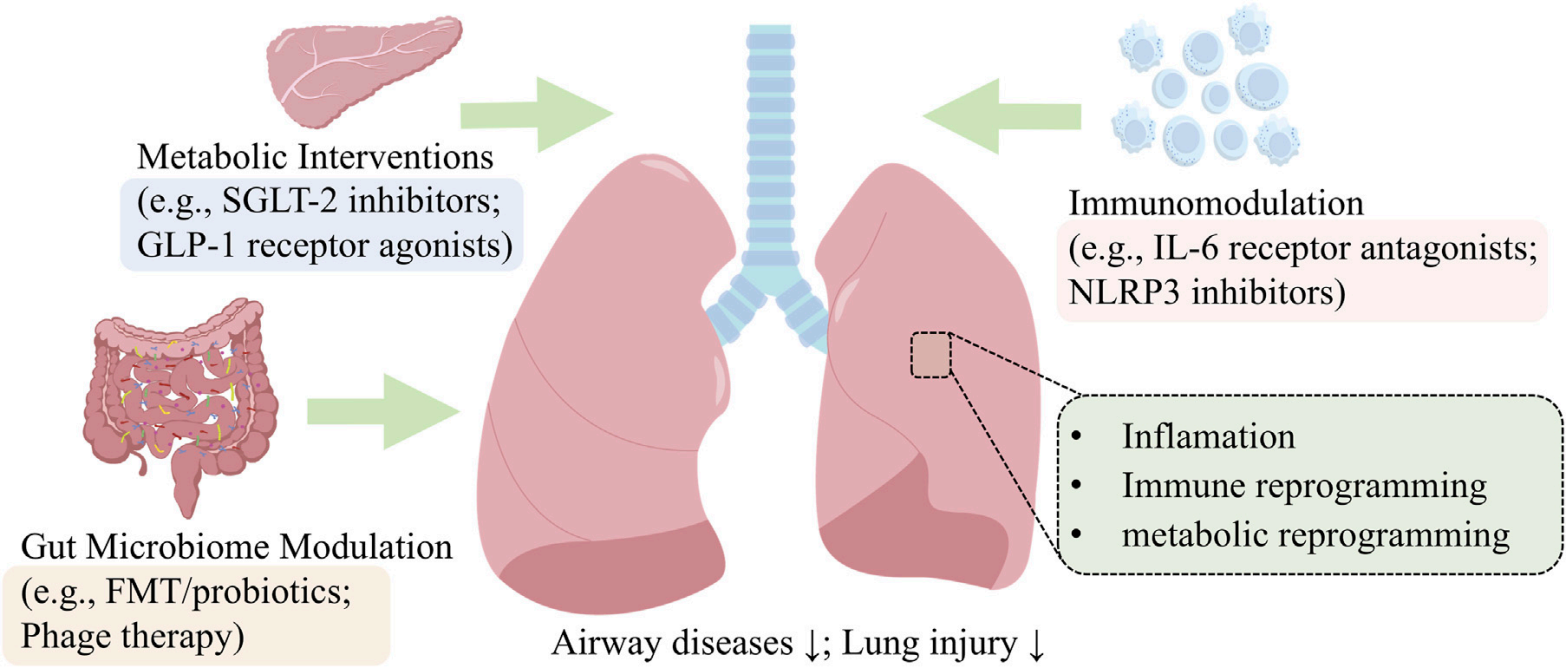
Therapeutic Targeting of the Metabolic-Microbial-Immune Axis. This schematic summarizes promising therapeutic strategies that pharmacologically target the metabolic (GLP-1RAs, SGLT-2is), microbial (FMT, phages), and immune (anti-IL-6, NLRP3) components of diabetic lung injury.

Having established the “metabolic dysregulation-gut dysbiosis-immune imbalance” axis as the core pathogenic mechanism, we now examine corresponding therapeutic strategies. Interventions targeting this axis—including GLP-1RAs/SGLT-2is, microbiota-directed therapy, and anti-IL-6/NLRP3 agents—hold significant promise (Figure 6). This axis provides the logical framework for the following review of current and emerging treatments, guiding the discussion of their translational potential and challenges.

### 7.1 Targeting shared metabolic-immune pathomechanisms

Emerging therapeutic paradigms in diabetes mellitus (DM) leverage the pleiotropic benefits of sodium-glucose cotransporter-2 inhibitors (SGLT-2Is) and glucagon-like peptide-1 receptor agonists (GLP-1RAs), extending beyond glycemic control to systemic immunomodulation. SGLT-2Is block renal glucose reabsorption to promote urinary excretion, while GLP-1RAs enhance glucose-dependent insulin secretion and suppress glucagon release (Prattichizzo et al., 2021). Both classes exhibit pulmonary protective effects *via* systemic anti-inflammatory reprogramming, with clinical studies demonstrating reduced exacerbation risk in T2DM patients with comorbid COPD (Foer et al., 2023). Therefore, SGLT-2Is and GLP-1RAs exemplify dual-pathway therapeutics, synergizing antihyperglycemic efficacy with systemic immunometabolic reprogramming to confer multi-organ protection, positioning these agents as cornerstone interventions for mitigating diabetes-associated pulmonary and systemic inflammatory comorbidities.

As previously discussed, metabolic imbalance and mitochondrial dysfunction under diabetic conditions are key mechanisms leading to lung injury. Therefore, restoring metabolic balance represents a promising therapeutic target. Beyond lowering blood glucose by promoting urinary glucose excretion, SGLT-2 inhibitors have been shown to activate the AMPK pathway (El-Horany et al., 2023), thereby improving mitochondrial function, alleviating endoplasmic reticulum stress and oxidative stress, reversing metabolic defects, and consequently protecting lung tissue. The prototypical inhibitor canagliflozin (CANA) attenuates pulmonary injury by skewing alveolar macrophage polarization toward tissue-reparative M2 phenotypes *via* PPARγ-dependent fatty acid oxidation (Lin et al., 2020). Concurrently, CANA restores mitochondrial homeostasis by activating the PINK1-Parkin mitophagy pathway while suppressing oxidative stress and endoplasmic reticulum stress (ERS), demonstrating efficacy across multiorgan injury models (O'Keefe et al., 2023; Packer, 2020). Although direct clinical evidence for their pulmonary protective effects is still accumulating, meta-analyses of large cardiovascular outcome trials indicate that SGLT-2 inhibitors significantly reduce the risk of acute exacerbations in diabetic patients with comorbid COPD (Foer et al., 2023), suggesting that they provide a pleiotropic organ protection beyond hypoglycemic effects.

GLP-1 receptor activation in sepsis models inhibits Toll-like receptor (TLR)-driven TNF-α production, mitigating systemic inflammation and acute lung injury (Wong et al., 2024). Clinically, GLP-1RAs reduce asthma exacerbations in DM patients, potentially by dampening allergen-induced airway neutrophilia (Lee et al., 2025; Toki et al., 2021). Both drug classes inhibit fibrotic progression and pulmonary inflammation by targeting key pathways—NF-κB signaling and NLRP3 inflammasome activation—thereby disrupting pro-inflammatory cytokine cascades (Shakour et al., 2024; Yang et al., 2022; Wang Y. et al., 2023). Therefore, CANA and GLP-1RAs orchestrate pleiotropic therapeutic benefits by converging on mitochondrial quality control, macrophage polarization, and inflammasome-cytokine axis suppression, thereby resolving immunometabolic crossfire in pulmonary systems and positioning these agents as cornerstone therapies for diabetes-related multiorgan inflammatory sequelae.

Emerging therapeutic strategies emphasize epigenetic precision therapeutics for DM-related tissue injury. Emerging strategies focus on microRNA (miR)-155, a master epigenetic regulator of DM-related multiorgan complications (Huang et al., 2014). The inhibition of miR-155 rescues redox homeostasis and mitochondrial dynamics in diabetic tissues *via* AMPKα-PGC1β signaling (Prieto et al., 2023; Ying et al., 2017), and suppresses alveolar macrophage M1 polarization in lung injury models by silencing the SOCS1/STAT1 axis, reducing neutrophil extracellular trap (NET) formation and IL-6/IL-1β-driven inflammation (Xu Y. et al., 2023). Antagomirs and CRISPR/Cas9-based editing systems enable tissue-specific miR-155 modulation, offering targeted therapeutic potential.

The dual targeting of immunometabolic pathways (*via* SGLT-2Is/GLP-1RAs) and epigenetic regulators (e.g., miR-155) represents a paradigm shift in DM therapeutics. These approaches address both systemic inflammation and tissue-specific damage, highlighting opportunities for precision medicine in diabetic complications. However, it must be noted that these pulmonary outcomes are largely derived from exploratory analyses or observational studies, and specifically designed prospective RCTs are warranted to confirm their efficacy against specific pulmonary diseases, such as idiopathic pulmonary fibrosis.

### 7.2 Metabolic reprogramming

First-line antidiabetic agents targeting AMPK and PPAR-γ exhibit potent immunomodulatory and cytoprotective effects through metabolic pathway modulation. As mentioned earlier, imbalance in the AMPK/mTOR energy-sensing pathway under diabetic conditions is a key mechanism driving metabolic stress and fibrotic transformation in alveolar epithelial cells. Consequently, restoring AMPK activity presents an attractive therapeutic target. Metformin, a canonical AMPK agonist, preserves airway epithelial integrity by stabilizing tight junctions (ZO-1/occludin) and restoring CFTR-mediated ion transport, reducing neutrophilic inflammation (Patkee et al., 2016; Myerburg et al., 2010). Furthermore, it exerts potent antifibrotic effects in pulmonary systems *via* TGF-β1/Smad3 inhibition, collagen I/III suppression, and PPAR-γ-driven adipogenic reprogramming of lung fibroblasts (evidenced by lipid droplet accumulation and FABP4 upregulation) (Kheirollahi et al., 2019). Pioglitazone, a PPAR-γ agonist, ameliorates pulmonary vascular dysfunction in hypertension by enhancing mitochondrial fatty acid oxidation and transcriptional remodeling (Legchenko et al., 2018). Furthermore, it restores immune equilibrium *via* PPAR-γ-dependent macrophage polarization and Treg/Th17 balance regulation (Carvalho et al., 2021; Nobs and Kopf, 2018). Therefore, by dual targeting of AMPK and PPAR-γ signaling, these first-line agents orchestrate pulmonary protection through mitochondrial bioenergetic restoration, epithelial barrier stabilization, and immunofibrotic balance, forging a metabolic-immunological nexus that mitigates diabetic pulmonary complications beyond conventional glycemic management. However, their efficacy and optimal dosing in human diabetic pulmonary fibrosis lack support from large-scale clinical data. Furthermore, future research needs to identify which patient subgroups are most likely to benefit from the pulmonary protective effects of these agents.

As noted earlier, aberrant glycogen metabolism in macrophages within the diabetic milieu leads to UDP-glucose (UDPG) accumulation. Binding and activation of the purinergic receptor P2Y14R by UDPG drives STAT1-dependent pro-inflammatory polarization, representing a key initiating mechanism for the inflammatory storm in diabetic lung injury (Ma et al., 2020). Therefore, P2Y14R emerges as a novel and highly potent target for directly interrupting this specific ‘metabolite-immune’ crosstalk for precise anti-inflammatory therapy. The development of new antagonists for this target has made preliminary progress. A recent study reported a highly potent P2Y14R antagonist, compound 25L, based on a 3-sulfonamidobenzoic acid scaffold. This compound demonstrated excellent properties in preclinical studies: its inhibitory activity against P2Y14R (IC_50_ = 5.6 nM) surpassed that of the lead compound PPTN, with more favorable pharmacokinetic characteristics (Ma et al., 2025). Crucially, in an LPS-induced mouse model of acute lung injury, treatment with compound 25L significantly alleviated pulmonary inflammatory infiltration and levels of pro-inflammatory cytokines (IL-1β, IL-6, TNF-α), providing direct pharmacological evidence supporting the hypothesis that P2Y14R inhibition alleviates lung inflammation (Ma et al., 2025). However, it must be clearly recognized that applying such P2Y14R antagonists to diabetic pulmonary injury faces significant challenges. All reported P2Y14R antagonists remain in the preclinical stage and have not entered clinical development. Common bottlenecks include: first, the need to confirm their long-term efficacy and safety in the context of chronic metabolic diseases such as diabetes, which differs significantly from acute injury models; second, the precise inhibition of the macrophage-specific UDPG-P2Y14R axis in the diabetic state by these compounds requires validation in disease models. Therefore, future research urgently needs to evaluate the efficacy of compound 25L and its analogs in diabetic animal models, focusing on resolving potential off-target effects and long-term toxicological issues to advance this promising target toward clinical translation.

Acetyl-CoA carboxylase (ACC) inhibition demonstrates therapeutic potential for metabolic syndrome by modulating lipid biosynthesis and energy homeostasis (Harwood, 2005). Concurrently, ACC inhibition reduces inflammatory macrophage recruitment through blockade of chemokine receptors CCR2/CCR5 (e.g., *via* cenicriviroc), thereby attenuating pro-inflammatory cytokine production in pulmonary niches (Tacke and Weiskirchen, 2018). Furthermore, ACC-targeted interventions enhance lipid metabolism in pulmonary macrophages, restricting intracellular lipid droplet availability and thereby reducing *Mycobacterium tuberculosis* survival and infection likelihood (Brandenburg et al., 2021). BAM15, a mitochondrial uncoupling agent, reverses diet-induced obesity and insulin resistance in murine models while enhancing mitochondrial bioenergetics through cristae remodeling and UCP1-independent thermogenesis (Alexopoulos et al., 2020; Axelrod et al., 2020). Furthermore, in sepsis models, BAM15 polarizes macrophages toward anti-inflammatory phenotypes and reduces pro-inflammatory cytokine production *via* AMPK-SIRT1-PGC1α axis activation (Dang et al., 2021). These multimodal mechanisms underscore BAM15’s therapeutic potential as a pharmacological candidate for metabolic-inflammatory disorders. However, it is important to note that mitochondrial uncouplers, due to their impact on systemic energy metabolism, may cause difficult-to-control side effects. Achieving lung-specific targeting while avoiding systemic adverse reactions is a prerequisite for developing them into viable drugs. Currently, the research focus in this area should be on developing lung-targeted delivery systems.

### 7.3 Microbiome-based targeted therapeutics

Metabolites resulting from gut dysbiosis (e.g., decreased SCFAs, increased BCAAs) and endotoxin (LPS) translocation exacerbate pulmonary inflammation *via* the gut-lung axis. Fecal microbiota transplantation (FMT) from healthy donors to diabetic recipients restores gut microbial diversity. FMT restores SCFA-producing bacteria (e.g., *Faecalibacterium*), thereby elevating SCFA levels and subsequently activating the GLP-1R axis to improve metabolism (de Groot et al., 2021; Ng et al., 2022; Tanase et al., 2020; Wu et al., 2022). Specific bacterial consortia (e.g., *Akkermansia muciniphila* + *Bifidobacterium longum*) further repair intestinal barrier integrity *via* P9 peptide secretion and AMPK-FOXO1 pathway activation, stabilizing postprandial glycemia (Yoon et al., 2021). Therefore, FMT-mediated restoration of gut microbial diversity activates SCFA-GLP-1 axis-driven metabolic reprogramming and bacterial consortia-enhanced barrier repair, synergistically ameliorating diabetic immunometabolic dyshomeostasis through microbiome-pancreatic crosstalk.

This review highlighted that metabolic endotoxemia resulting from gut dysbiosis (e.g., LPS translocation) is a core event driving pulmonary hyperinflammation *via* the TLR4/NF-κB pathway. Precision modulation of the gut microbiota, particularly reducing LPS-producing Gram-negative pathogens, represents a potential therapeutic target. Against this backdrop, targeted phage therapy demonstrates unique promise. The gut virome of individuals with DM exhibits characteristic alterations, including an enrichment of *Caudovirales* phages, which enhance antibiotic resistance in pathogens (e.g., *Klebsiella pneumoniae*) *via* horizontal gene transfer. Targeted phage cocktail therapy achieves a reduction in pathogenic bacterial load while preserving commensal microbiota stability (Nishijima et al., 2022; Hu et al., 2023; Shkoporov et al., 2022). Gut-derived phages cross the intestinal barrier *via* receptor-mediated transcytosis and migrate to pulmonary tissues through the portal venous system, enabling tissue-specific colonization (Ling et al., 2023; Mousavinasab et al., 2023; Naveen Kumar et al., 2025). The pH-responsive microcapsules enable site-specific intestinal release of encapsulated phages, followed by systemic circulation-mediated targeting to inflamed pulmonary niches. Integration of phage pharmacokinetic modeling with real-time host metabolomic profiling enables dynamic assessment of gut-lung axis modulation, allowing iterative refinement of personalized regimens. However, such therapies are currently largely confined to preclinical proof-of-concept. Translating this strategy from the laboratory to the clinic faces numerous severe challenges. As exogenous proteins, the inherent immunogenicity of phages and their colonization stability in humans remain largely unknown. Therefore, future research must prioritize addressing the challenges of precise phage delivery, *in vivo* kinetics, and strategies to evade host immune clearance.

### 7.4 Inflammatory network regulation and precision therapy

Although PGE2 is a classic inflammatory mediator, its downstream signaling pathways through different receptors (such as EP4) may be precisely regulated to achieve anti-inflammatory and tissue repair effects. PGE2-EP4 activation elevates cAMP, upregulating 15-lipoxygenase (15-LOX) to shift lipid mediator production from pro-inflammatory leukotriene B4 (LTB4) to anti-inflammatory lipoxin A4 (LXA4). This metabolic switch suppresses neutrophil pulmonary infiltration by downregulating CXCR2 expression and inhibiting NADPH oxidase assembly, reducing bronchoalveolar lavage fluid (BALF) protein extravasation in septic models (Jiao et al., 2023). During pulmonary recovery, PGE2-EP2/EP4 signaling activates CREB, epigenetically enhancing PD-1 expression in lung tissue-resident memory T (TRM) cells. This primes TRM cells for rapid pathogen response while maintaining immune homeostasis (Feehan et al., 2024). These findings collectively demonstrate the critical role of PGE2 in orchestrating long-term immune dynamics and suggest that precision-targeted modulation of its inflammatory pathways—through spatiotemporal regulation of EP receptor signaling—may confer dual therapeutic benefits for both diabetic complications and pulmonary injury.

Emerging study demonstrates that targeted delivery of nanomedicines disrupts inflammatory signaling pathways commonly associated with DM and pulmonary injury, thereby achieving therapeutic efficacy. As described above, the activation of NLRP3 inflammasome leads to the maturation of IL-1β and IL-18, driving lung inflammation. Through structural optimization, the orally administered inhibitor DFV890 achieves colon-specific release, suppressesing ASC oligomerization and IL-1β maturation in diabetic lung injury models. It can enter enterohepatic recirculation to sustain pyroptosis pathway inhibition, offering durable anti-inflammatory effects (Gatlik et al., 2024). Although DFV890, as an oral NLRP3 inhibitor, has shown good tolerability in initial human trials, this represents only the first step in its clinical translation. Key questions remain regarding its ability to effectively inhibit NLRP3 inflammasome activation within the complex milieu of diabetic lung injury, and whether long-term suppression of this core innate immune pathway might lead to the activation of compensatory inflammatory pathways or other unknown off-target effects, all of which require longer-term clinical investigation.

Within the complex inflammatory network of diabetic pulmonary injury described earlier, IL-6 has been identified as a key cytokine driving the acute phase response and immune cell infiltration. Therefore, directly blocking the IL-6 signaling pathway is a rational strategy for controlling aberrant pulmonary inflammation. Tocilizumab, a humanized monoclonal antibody against the IL-6 receptor, is re-evaluated in this context. Tocilizumab is innovatively encapsulated in pH-sensitive nanoparticles, enabling site-specific release at inflammatory loci (pH < 6.5), which enhances pulmonary soluble IL-6 receptor (sIL-6R) neutralization efficiency. In clinical studies of diabetes with comorbid interstitial lung disease, tocilizumab effectively slowed the decline in diffusing capacity for carbon monoxide, potentially by neutralizing IL-6 and breaking the inflammatory cycle (Tanaka et al., 2018; Khanna et al., 2022). Case reports have documented its therapeutic application in managing post-pulmonary infection complications in elderly diabetic patients (Kai et al., 2022). This suggests that combining validated biologics with advanced delivery technologies represents a future direction for precision immunomodulation. However, caution is warranted as the IL-6 pathway plays a crucial role in host defense; thus, long-term use may increase the risk of serious infections. For diabetic patients who are susceptible to themselves, such risks need to be highly valued in future risk-benefit assessments.

In diabetes, Treg cells exhibit a functional impairment that compromises their immunosuppressive capacity. CRISPR/dCas9-mediated precision editing of the FOXP3 enhancer region generates stabilized Treg cells enhanced suppressive activity *in vitro*. Adoptive transfer of these cells in diabetic pulmonary injury models reduced neutrophil proportion in bronchoalveolar lavage fluid, while maintaining antimicrobial defense capacity (Esensten et al., 2018; Ferreira et al., 2019). A thermosensitive hydrogel loaded with IL-2/JES6-1 complexes enables inflammation-triggered sustained release in pulmonary microenvironments, enhancing Treg recruitment efficiency at lesion sites. This strategy demonstrated greater improvement in PaO2/FiO2 ratio compared to conventional therapies in graft-versus-host disease-associated pulmonary injury (Esensten et al., 2018; Ferreira et al., 2019; Wang C. et al., 2021). Thus, adoptive transfer of engineered Treg cells provides a precise tool for restoring immune tolerance. However, the high technical complexity and cost of this approach severely limit its broad application. More critically, safety remains a paramount hurdle: Are the edited Treg cells stable *in vivo*? Is there a risk of their conversion into pathogenic effector cells? Before considering any clinical application, stringent safety guidelines must be established, and long-term follow-up studies in larger animal models are imperative.

#### 7.4.1 Summary and prospect

Emerging mechanistic insights into DM-associated pulmonary injury have unveiled a sophisticated multi-axis interplay among metabolic regulatory networks, gut microbial homeostasis, and host immune response systems. This field necessitates comprehensive investigations from a tripartite metabolic-microbial-immune interaction perspective to systematically elucidate the pathogenesis, comorbidity progression patterns, and prognostic determinants of diabetic pulmonary complications. Clinical epidemiological studies have established significant comorbidities between DM and respiratory disorders including lung cancer and COPD. The metabolic regulatory network integrates nutrient-sensing pathways encompassing glucose metabolism, lipid homeostasis, and amino acid flux, forming the pivotal pathological nexus linking DM to its pulmonary complications. Mechanistically, DM-induced perturbations in the AMPK/mTOR signaling axis, impaired branched-chain amino acid catabolism, and tryptophan-kynurenine axis dysregulation collectively drive oxidative stress and immune homeostasis disruption through cascade reactions, culminating in pulmonary parenchymal injury. Crucially, gut microbiota exerts central regulatory control over diabetic pulmonary injury progression *via* bidirectional gut-lung axis crosstalk. Operative mechanisms include: 1) Immune network remodeling mediated by microbiota-derived metabolites; 2) Synergistic amplification of endogenous metabolic disturbances and exogenous environmental stressors. Under pathological conditions such as endotoxemia and intestinal barrier dysfunction, dysbiosis-driven metabolic reprogramming converts entero-derived metabolic signals into pro-inflammatory cytokine cascades, thereby potentiating pulmonary inflammatory microenvironment formation.

#### 7.4.2 Emerging therapeutic dilemmas in DM-pulmonary comorbidity management

While the causal relationship between DM and pulmonary disorders remains incompletely elucidated, the clinical management of their comorbid status presents emerging challenges. Shared inflammatory pathophysiology and multisystem interactions substantially amplify clinical complexity, particularly evident in diabetic patients with concurrent COPD. Current practice reveals a critical therapeutic paradox: although international guidelines endorse inhaled corticosteroids (ICS) as standard COPD therapy (Agustí et al., 2023), their dose-dependent induction of glycemic variability (Slatore et al., 2009) establishes potentially conflicting treatment paradigms.

Dual Therapeutic Benefits of GLP-1 Receptor Agonists: Glucagon-like peptide-1 (GLP-1), an incretin hormone secreted by intestinal L-cells, activates cAMP/PKA signaling through GLP-1 receptor (GLP-1R) engagement, not only potentiating glucose-dependent insulin secretion for glycemic control (Kim and Egan, 2008) but also exerting novel respiratory effects. Clinical evidence demonstrates that GLP-1 receptor agonists (GLP-1RAs) confer dual benefits in DM-COPD comorbidity: reducing asthma exacerbation risk (Foer et al., 2023), while ameliorating bronchial hyperresponsiveness *via* PKA activation in airway epithelial cells (Rogliani et al., 2016). Mechanistically, these agents remodel alveolar macrophage metabolic phenotypes and suppress NF-κB-mediated inflammatory cascades.

#### 7.4.3 Future directions in metabo-inflammatory modulation

These breakthroughs underscore the therapeutic potential of targeting metabo-inflammatory crosstalk for multidimensional comorbidity management (Table 1). Advancing this frontier requires establishing multi-omics predictive models to optimize personalized dosing regimens within evidence-based frameworks, thereby achieving synergistic glycemic and pulmonary functional outcomes.

**TABLE 1:** Multidimensional therapeutic strategies targeting diabetic pulmonary injury.

Therapeutic target	Mechanism of action	Representative agents/Interventions	Clinical evidence
Metabolic interventions			
SGLT-2 inhibitors	Inhibit glucose reabsorption; reduce oxidative/ER stress; modulate macrophage polarization	Canagliflozin, Dapagliflozin	Reduces COPD exacerbation risk; attenuates sepsis-induced lung injury (Lin et al., 2020; O'Keefe et al., 2023; Packer, 2020)
GLP-1 receptor agonists	Stimulate insulin secretion; suppress TLR-mediated TNF-α release; regulate neutrophil recruitment	Liraglutide, Semaglutide	Decreases asthma incidence; inhibits pulmonary fibrosis (Wong et al., 2024; Lee et al., 2025; Toki et al., 2021)
AMPK agonists	Maintain airway epithelial integrity; suppress TGF-β1-driven fibrosis	Metformin	Anti-fibrotic effects; enhances infection resistance (Patkee et al., 2016; Myerburg et al., 2010; Kheirollahi et al., 2019)
PPAR-γ agonists	Regulate fatty acid oxidation; improve mitochondrial function; suppress pulmonary inflammation	Pioglitazone	Modulates lung immune development; improves hypertensive vascular function (Legchenko et al., 2018; Carvalho et al., 2021; Nobs and Kopf, 2018)
P2Y14R antagonists	Suppress pulmonary inflammation	Compound 25L	Reduced the inflammatory response and the levels of proinflammatory cytokines in lung tissues (Ma et al., 2025)
ACC inhibitors	Block CCR2/CCR5-dependent inflammatory macrophage recruitment; enhance lipid metabolism	Cenicriviroc	Reduces tuberculosis susceptibility (Harwood, 2005; Tacke and Weiskirchen, 2018; Brandenburg et al., 2021)
Mitochondrial uncouplers	Reverse insulin resistance; enhance mitochondrial efficiency; promote anti-inflammatory macrophages	BAM15	Mitigates sepsis-associated inflammation (Alexopoulos et al., 2020; Axelrod et al., 2020; Dang et al., 2021)
Gut microbiome modulation			
FMT/probiotics	Restore microbial diversity; reverse gut hyperpermeability; balance SCFA/BCAA metabolism	Human-derived probiotics (e.g., *Bifidobacterium*)	Improves insulin sensitivity; reduces systemic inflammation (de Groot et al., 2021; Ng et al., 2022; Tanase et al., 2020; Wu et al., 2022)
Phage therapy	Precisely edit gut virome; block gut-lung axis transmission of LPS	CRISPR-engineered phages	Regulates pulmonary immune responses (Ling et al., 2023; Mousavinasab et al., 2023)
Immunomodulation			
PGE2 pathway modulators	Shift lipid mediator profile (LTB4→LXA4); inhibit neutrophil recruitment/NET formation	EP4 receptor agonists	Controls sepsis-induced injury; enhances T cell memory (Jiao et al., 2023; Feehan et al., 2024)
NLRP3 inhibitors	Block inflammasome activation; reduce IL-1β release	DFV890	Specifically targets NLRP3 signaling (Gatlik et al., 2024)
IL-6 receptor antagonists	Neutralize IL-6 signaling; suppress systemic inflammation	Tocilizumab	Effective in interstitial lung disease; treats diabetes-associated pulmonary infection (Tanaka et al., 2018; Khanna et al., 2022; Kai et al., 2022)
Treg cell therapy	Re-establish immune tolerance; suppress hyperinflammation	Exogenous Treg infusion	Demonstrates safety in type 1 diabetes and transplantation (Esensten et al., 2018; Ferreira et al., 2019)
Epigenetic modulation			
miRNA regulators	Inhibit pro-inflammatory miRNAs (e.g., miR-155); reduce M1 macrophage polarization	miRNA antagonists (e.g., antagomir-155)	Attenuates lung injury-associated inflammation (Huang et al., 2014; Prieto et al., 2023; Ying et al., 2017; Xu et al., 2023b)

Persisting knowledge gaps surround the mechanistic contributions of dysregulated metabolic functions to disease pathogenesis. Groundbreaking advances in metabolomics and metagenomics have enabled integrated multi-omics frameworks to systematically deconvolute the multidimensional “metabolism-microbiota-immune” interactome. This field is undergoing a paradigm shift—wherein cross-scale biological network modeling allows precise identification of critical regulatory nodes governing metabolic imbalance and immune dysregulation. Such systems biology perspectives not only drive fundamental understanding of comorbidity mechanisms but also provide a theoretical foundation for developing next-generation trans-organ therapeutic strategies targeting systemic immunometabolic homeostasis.

## References

[B1] Agustí A. Celli B. R. Criner G. J. Halpin D. Anzueto A. Barnes P. (2023). Global initiative for chronic obstructive lung disease 2023 report: GOLD executive summary. Eur. Respir. J. 61 (4), 2300239. 10.1183/13993003.00239-2023 36858443 PMC10066569

[B2] Ahmad N. Srinivasan K. Moudgil H. (2011). Diabetes and lung function: part of a wider spectrum. Chest 139 (1), 235–236. 10.1378/chest.10-2095 21208894

[B3] Alexopoulos S. J. Chen S. Y. Brandon A. E. Salamoun J. M. Byrne F. L. Garcia C. J. (2020). Mitochondrial uncoupler BAM15 reverses diet-induced obesity and insulin resistance in mice. Nat. Commun. 11 (1), 2397. 10.1038/s41467-020-16298-2 32409697 PMC7224297

[B4] Avellaneda-Franco L. Xie L. Nakai M. Barr J. J. Marques F. Z. (2024). Dietary fiber intake impacts gut bacterial and viral populations in a hypertensive mouse model. Gut microbes 16 (1), 2407047. 10.1080/19490976.2024.2407047 39340212 PMC11567275

[B5] Axelrod C. L. King W. T. Davuluri G. Noland R. C. Hall J. Hull M. (2020). BAM15-mediated mitochondrial uncoupling protects against obesity and improves glycemic control. EMBO Mol. Med. 12 (7), e12088. 10.15252/emmm.202012088 32519812 PMC7338798

[B6] Betteridge D. J. (2000). What is oxidative stress? Metabolism Clin. Exp. 49 (2 Suppl. 1), 3–8. 10.1016/s0026-0495(00)80077-3 10693912

[B7] Blair M. C. Neinast M. D. Jang C. Chu Q. Jung J. W. Axsom J. (2023). Branched-chain amino acid catabolism in muscle affects systemic BCAA levels but not insulin resistance. Nat. Metab. 5 (4), 589–606. 10.1038/s42255-023-00794-y 37100997 PMC10278155

[B8] Bonyek-Silva I. Machado A. F. A. Cerqueira-Silva T. Nunes S. Silva Cruz M. R. Silva J. (2021). LTB(4)-Driven inflammation and increased expression of ALOX5/ACE2 during severe COVID-19 in individuals with diabetes. Diabetes 70 (9), 2120–2130. 10.2337/db20-1260 34417262 PMC8576416

[B9] Brandenburg J. Marwitz S. Tazoll S. C. Waldow F. Kalsdorf B. Vierbuchen T. (2021). WNT6/ACC2-induced storage of triacylglycerols in macrophages is exploited by *Mycobacterium tuberculosis* . J. Clin. investigation 131 (16), e141833. 10.1172/JCI141833 34255743 PMC8363280

[B10] Brown E. J. Albers M. W. Shin T. B. Ichikawa K. Keith C. T. Lane W. S. (1994). A Mammalian protein targeted by G1-arresting rapamycin-receptor complex. Nature 369 (6483), 756–758. 10.1038/369756a0 8008069

[B11] Burcelin R. (2016). Gut microbiota and immune crosstalk in metabolic disease. Mol. Metab. 5 (9), 771–781. 10.1016/j.molmet.2016.05.016 27617200 PMC5004167

[B12] Burgy O. Loriod S. Beltramo G. Bonniaud P. (2022). Extracellular lipids in the lung and their role in pulmonary fibrosis. Cells 11 (7), 1209. 10.3390/cells11071209 35406772 PMC8997955

[B13] Canfora E. E. Meex R. C. R. Venema K. Blaak E. E. (2019). Gut microbial metabolites in obesity, NAFLD and T2DM. Nat. Rev. Endocrinol. 15 (5), 261–273. 10.1038/s41574-019-0156-z 30670819

[B14] Cani P. D. Amar J. Iglesias M. A. Poggi M. Knauf C. Bastelica D. (2007). Metabolic endotoxemia initiates obesity and insulin resistance. Diabetes 56 (7), 1761–1772. 10.2337/db06-1491 17456850

[B15] Carvalho M. V. Gonçalves-de-Albuquerque C. F. Silva A. R. (2021). PPAR gamma: from definition to molecular targets and therapy of lung diseases. Int. J. Mol. Sci. 22 (2), 805. 10.3390/ijms22020805 33467433 PMC7830538

[B16] Casas S. Novials A. Reimann F. Gomis R. Gribble F. M. (2008). Calcium elevation in mouse pancreatic beta cells evoked by extracellular human islet amyloid polypeptide involves activation of the mechanosensitive ion channel TRPV4. Diabetologia 51 (12), 2252–2262. 10.1007/s00125-008-1111-z 18751967 PMC7212067

[B17] Castro E. C. Sen P. Parks W. T. Langston C. Galambos C. (2017). The role of serotonin transporter in human lung development and in neonatal lung disorders. Can. Respir. J. 2017, 9064046. 10.1155/2017/9064046 28316463 PMC5337869

[B18] Caughey G. E. Roughead E. E. Vitry A. I. McDermott R. A. Shakib S. Gilbert A. L. (2010). Comorbidity in the elderly with diabetes: identification of areas of potential treatment conflicts. Diabetes Res. Clin. Pract. 87 (3), 385–393. 10.1016/j.diabres.2009.10.019 19923032

[B19] Caughey G. E. Preiss A. K. Vitry A. I. Gilbert A. L. Roughead E. E. (2013). Comorbid diabetes and COPD: impact of corticosteroid use on diabetes complications. Diabetes care 36 (10), 3009–3014. 10.2337/dc12-2197 23735725 PMC3781532

[B20] Chávez-Talavera O. Tailleux A. Lefebvre P. Staels B. (2017). Bile acid control of metabolism and inflammation in obesity, type 2 diabetes, dyslipidemia, and nonalcoholic Fatty liver disease. Gastroenterology 152 (7), 1679–1694.e1673. 10.1053/j.gastro.2017.01.055 28214524

[B21] Chen Y. Zhang F. Wang D. Li L. Si H. Wang C. (2020). Mesenchymal stem cells attenuate diabetic lung fibrosis *via* adjusting Sirt3-Mediated stress responses in rats. Oxidative Med. Cell. Longev. 2020, 8076105. 10.1155/2020/8076105 32089781 PMC7024095

[B22] Cheng K. T. Xiong S. Ye Z. Hong Z. Di A. Tsang K. M. (2017). Caspase-11-mediated endothelial pyroptosis underlies endotoxemia-induced lung injury. J. Clin. investigation 127 (11), 4124–4135. 10.1172/JCI94495 28990935 PMC5663346

[B23] Chiou J. Geusz R. J. Okino M. L. Han J. Y. Miller M. Melton R. (2021). Interpreting type 1 diabetes risk with genetics and single-cell epigenomics. Nature 594 (7863), 398–402. 10.1038/s41586-021-03552-w 34012112 PMC10560508

[B24] Cho N. H. Shaw J. E. Karuranga S. Huang Y. da Rocha Fernandes J. D. Ohlrogge A. W. (2018). IDF Diabetes Atlas: global estimates of diabetes prevalence for 2017 and projections for 2045. Diabetes Res. Clin. Pract. 138, 271–281. 10.1016/j.diabres.2018.02.023 29496507

[B25] Conroy L. R. Clarke H. A. Allison D. B. Valenca S. S. Sun Q. Hawkinson T. R. (2023). Spatial metabolomics reveals glycogen as an actionable target for pulmonary fibrosis. Nat. Commun. 14 (1), 2759. 10.1038/s41467-023-38437-1 37179348 PMC10182559

[B26] Conte C. Cipponeri E. Roden M. (2024). Diabetes Mellitus, energy metabolism, and COVID-19. Endocr. Rev. 45 (2), 281–308. 10.1210/endrev/bnad032 37934800 PMC10911957

[B27] Dang C. P. Issara-Amphorn J. Charoensappakit A. Udompornpitak K. Bhunyakarnjanarat T. Saisorn W. (2021). BAM15, a mitochondrial uncoupling agent, attenuates inflammation in the LPS Injection Mouse model: an adjunctive anti-inflammation on macrophages and hepatocytes. J. innate Immun. 13 (6), 359–375. 10.1159/000516348 34062536 PMC8613553

[B28] Darby W. G. Grace M. S. Baratchi S. McIntyre P. (2016). Modulation of TRPV4 by diverse mechanisms. Int. J. Biochem. and Cell. Biol. 78, 217–228. 10.1016/j.biocel.2016.07.012 27425399

[B29] De Giovanni M. Dang E. V. Chen K. Y. An J. Madhani H. D. Cyster J. G. (2023). Platelets and mast cells promote pathogenic eosinophil recruitment during invasive fungal infection *via* the 5-HIAA-GPR35 ligand-receptor system. Immunity 56 (7), 1548–1560.e5. 10.1016/j.immuni.2023.05.006 37279752 PMC10360074

[B30] de Groot P. Nikolic T. Pellegrini S. Sordi V. Imangaliyev S. Rampanelli E. (2021). Faecal microbiota transplantation halts progression of human new-onset type 1 diabetes in a randomised controlled trial. Gut 70 (1), 92–105. 10.1136/gutjnl-2020-322630 33106354 PMC7788262

[B31] de Vos W. M. Tilg H. Van Hul M. Cani P. D. (2022). Gut microbiome and health: mechanistic insights. Gut 71 (5), 1020–1032. 10.1136/gutjnl-2021-326789 35105664 PMC8995832

[B32] Dewanjee S. Chakraborty P. Bhattacharya H. Chacko L. Singh B. Chaudhary A. (2022). Altered glucose metabolism in Alzheimer's disease: role of mitochondrial dysfunction and oxidative stress. Free Radic. Biol. and Med. 193 (Pt 1), 134–157. 10.1016/j.freeradbiomed.2022.09.032 36206930

[B33] Du K. Sun L. Luo Z. Cao Y. Sun Q. Zhang K. (2022). Reduced DMPC and PMPC in lung surfactant promote SARS-CoV-2 infection in obesity. Metabolism Clin. Exp. 131, 155181. 10.1016/j.metabol.2022.155181 35311662 PMC8930181

[B34] Eid S. Sas K. M. Abcouwer S. F. Feldman E. L. Gardner T. W. Pennathur S. (2019). New insights into the mechanisms of diabetic complications: role of lipids and lipid metabolism. Diabetologia 62 (9), 1539–1549. 10.1007/s00125-019-4959-1 31346658 PMC6679814

[B35] Eid S. A. Rumora A. E. Beirowski B. Bennett D. L. Hur J. Savelieff M. G. (2023). New perspectives in diabetic neuropathy. Neuron 111 (17), 2623–2641. 10.1016/j.neuron.2023.05.003 37263266 PMC10525009

[B36] El-Horany H. E. Atef M. M. Abdel Ghafar M. T. Fouda M. H. Nasef N. A. Hegab I. I. (2023). Empagliflozin ameliorates bleomycin-induced pulmonary fibrosis in rats by modulating Sesn2/AMPK/Nrf2 signaling and targeting ferroptosis and autophagy. Int. J. Mol. Sci. 24 (11), 9481. 10.3390/ijms24119481 37298433 PMC10253289

[B37] Entezari M. Hashemi D. Taheriazam A. Zabolian A. Mohammadi S. Fakhri F. (2022). AMPK signaling in diabetes mellitus, insulin resistance and diabetic complications: a pre-clinical and clinical investigation. Biomed. and Pharmacother. = Biomedecine and Pharmacother. 146, 112563. 10.1016/j.biopha.2021.112563 35062059

[B38] Erener S. (2020). Diabetes, infection risk and COVID-19. Mol. Metab. 39, 101044. 10.1016/j.molmet.2020.101044 32585364 PMC7308743

[B39] Esensten J. H. Muller Y. D. Bluestone J. A. Tang Q. (2018). Regulatory T-cell therapy for autoimmune and autoinflammatory diseases: the next frontier. J. allergy Clin. Immunol. 142 (6), 1710–1718. 10.1016/j.jaci.2018.10.015 30367909

[B40] Fan G. Cao F. Kuang T. Yi H. Zhao C. Wang L. (2023). Alterations in the gut virome are associated with type 2 diabetes and diabetic nephropathy. Gut microbes 15 (1), 2226925. 10.1080/19490976.2023.2226925 37349979 PMC10291934

[B41] Feehan K. T. Bridgewater H. E. Stenkiewicz-Witeska J. De Maeyer R. P. H. Ferguson J. Mack M. (2024). Post-resolution macrophages shape long-term tissue immunity and integrity in a mouse model of pneumococcal pneumonia. Nat. Commun. 15 (1), 4326. 10.1038/s41467-024-48138-y 38773113 PMC11109210

[B42] Fehrenbach H. Kasper M. Tschernig T. Shearman M. S. Schuh D. Müller M. (1998). **Receptor for advanced glycation endproducts (RAGE) exhibits highly differential cellular and subcellular localisation in rat and human lung**. *Cellular and molecular biology (Noisy-le-Grand, France)* . Cell. Mol. Biol. 44 (7), 1147–1157. 9846897

[B43] Fernández-Real J. M. Chico B. Shiratori M. Nara Y. Takahashi H. Ricart W. (2008). Circulating surfactant protein A (SP-A), a marker of lung injury, is associated with insulin resistance. Diabetes care 31 (5), 958–963. 10.2337/dc07-2173 18285549

[B44] Fernández-Real J. M. Valdés S. Manco M. Chico B. Botas P. Campo A. (2010). Surfactant protein d, a marker of lung innate immunity, is positively associated with insulin sensitivity. Diabetes care 33 (4), 847–853. 10.2337/dc09-0542 20086254 PMC2845040

[B45] Ferreira L. M. R. Muller Y. D. Bluestone J. A. Tang Q. (2019). Next-generation regulatory T cell therapy. Nat. Rev. Drug Discov. 18 (10), 749–769. 10.1038/s41573-019-0041-4 31541224 PMC7773144

[B46] Fisher A. B. (1984). Intermediary metabolism of the lung. Environ. health Perspect. 55, 149–158. 10.1289/ehp.8455149 6376097 PMC1568362

[B47] Foer D. Strasser Z. H. Cui J. Cahill K. N. Boyce J. A. Murphy S. N. (2023). Association of GLP-1 receptor agonists with chronic obstructive pulmonary disease exacerbations among patients with type 2 diabetes. Am. J. Respir. Crit. care Med. 208 (10), 1088–1100. 10.1164/rccm.202303-0491OC 37647574 PMC10867930

[B48] Fonseca W. Malinczak C. A. Fujimura K. Li D. McCauley K. Li J. (2021). Maternal gut microbiome regulates immunity to RSV infection in offspring. J. Exp. Med. 218 (11), e20210235. 10.1084/jem.20210235 34613328 PMC8500238

[B49] Forrester S. J. Kikuchi D. S. Hernandes M. S. Xu Q. Griendling K. K. (2018). Reactive oxygen species in metabolic and inflammatory signaling. Circulation Res. 122 (6), 877–902. 10.1161/CIRCRESAHA.117.311401 29700084 PMC5926825

[B50] Gao Z. Yin J. Zhang J. Ward R. E. Martin R. J. Lefevre M. (2009). Butyrate improves insulin sensitivity and increases energy expenditure in mice. Diabetes 58 (7), 1509–1517. 10.2337/db08-1637 19366864 PMC2699871

[B51] Gao P. Uzun Y. He B. Salamati S. E. Coffey J. K. M. Tsalikian E. (2019). Risk variants disrupting enhancers of T(H)1 and T(REG) cells in type 1 diabetes. Proc. Natl. Acad. Sci. U. S. A. 116 (15), 7581–7590. 10.1073/pnas.1815336116 30910956 PMC6462079

[B52] Gao J. Yang T. Song B. Ma X. Ma Y. Lin X. (2023). Abnormal tryptophan catabolism in diabetes mellitus and its complications: opportunities and challenges. Biomed. and Pharmacother. = Biomedecine and Pharmacother. 166, 115395. 10.1016/j.biopha.2023.115395 37657259

[B53] Gatlik E. Mehes B. Voltz E. Sommer U. Tritto E. Lestini G. (2024). First-in-human safety, tolerability, and pharmacokinetic results of DFV890, an oral low-molecular-weight NLRP3 inhibitor. Clin. Transl. Sci. 17 (5), e13789. 10.1111/cts.13789 38761014 PMC11101992

[B54] Goel K. Beatman E. L. Egersdorf N. Scruggs A. Cao D. Berdyshev E. V. (2021). Sphingosine 1 phosphate (S1P) receptor 1 is decreased in human lung Microvascular endothelial cells of smokers and mediates S1P effect on autophagy. Cells 10 (5), 1200. 10.3390/cells10051200 34068927 PMC8156252

[B55] Gojda J. Cahova M. (2021). Gut microbiota as the link between elevated BCAA serum levels and insulin resistance. Biomolecules 11 (10), 1414. 10.3390/biom11101414 34680047 PMC8533624

[B56] Golay A. Felber J. P. Meyer H. U. Curchod B. Maeder E. Jéquier E. (1984). Study on lipid metabolism in obesity diabetes. Metabolism Clin. Exp. 33 (2), 111–116. 10.1016/0026-0495(84)90121-5 6694554

[B57] Goncharova E. A. (2013). mTOR and vascular remodeling in lung diseases: current challenges and therapeutic prospects. FASEB J. official Publ. Fed. Am. Soc. Exp. Biol. 27 (5), 1796–1807. 10.1096/fj.12-222224 23355268 PMC3633815

[B58] Greer R. L. Morgun A. Shulzhenko N. (2013). Bridging immunity and lipid metabolism by gut microbiota. J. allergy Clin. Immunol. 132 (2), 253–262. 10.1016/j.jaci.2013.06.025 23905915

[B59] Guo W. A. Knight P. R. Raghavendran K. (2012). The receptor for advanced glycation end products and acute lung injury/acute respiratory distress syndrome. Intensive care Med. 38 (10), 1588–1598. 10.1007/s00134-012-2624-y 22777515

[B60] Haghikia A. Jörg S. Duscha A. Berg J. Manzel A. Waschbisch A. (2015). Dietary fatty acids directly impact central nervous System autoimmunity *via* the small intestine. Immunity 43 (4), 817–829. 10.1016/j.immuni.2015.09.007 26488817

[B61] Han S. Mallampalli R. K. (2015). The role of surfactant in lung disease and host defense against pulmonary infections. Ann. Am. Thorac. Soc. 12 (5), 765–774. 10.1513/AnnalsATS.201411-507FR 25742123 PMC4418337

[B62] Harayama T. Eto M. Shindou H. Kita Y. Otsubo E. Hishikawa D. (2014). Lysophospholipid acyltransferases mediate phosphatidylcholine diversification to achieve the physical properties required *in vivo* . Cell. metab. 20 (2), 295–305. 10.1016/j.cmet.2014.05.019 24981836

[B63] Harwood H. J. Jr. (2005). Treating the metabolic syndrome: acetyl-coa carboxylase inhibition. Expert Opin. Ther. targets 9 (2), 267–281. 10.1517/14728222.9.2.267 15934915

[B64] Hassan M. H. Galal O. Sakhr H. M. Kamaleldeen E. B. Zekry N. F. Fateen E. (2023). Profile of plasma free amino acids, carnitine and acylcarnitines, and JAK2(v617f) mutation as potential metabolic markers in children with type 1 diabetic nephropathy. Biomed. Chromatogr. BMC 37 (12), e5747. 10.1002/bmc.5747 37728037

[B65] Hatlen P. Grønberg B. H. Langhammer A. Carlsen S. M. Amundsen T. (2011). Prolonged survival in patients with lung cancer with diabetes mellitus. J. Thorac. Oncol. official Publ. Int. Assoc. Study Lung Cancer 6 (11), 1810–1817. 10.1097/JTO.0b013e31822a75be 21964531

[B66] He M. Y. Zhang P. Shi N. Li T. Wang J. He L. (2023). Nrf2 is involved in hyperglycemia-induced abnormal lung development through both antioxidation-dependent and antioxidation-independent activation. Am. J. Respir. Cell. Mol. Biol. 69 (2), 197–209. 10.1165/rcmb.2022-0345OC 36780671

[B67] Hetz C. Papa F. R. (2018). The unfolded protein response and cell fate control. Mol. Cell. 69 (2), 169–181. 10.1016/j.molcel.2017.06.017 29107536

[B68] Hsu H. S. Liu C. C. Lin J. H. Hsu T. W. Hsu J. W. Su K. (2017). Involvement of ER stress, PI3K/AKT activation, and lung fibroblast proliferation in bleomycin-induced pulmonary fibrosis. Sci. Rep. 7 (1), 14272. 10.1038/s41598-017-14612-5 29079731 PMC5660192

[B69] Hu J. Ding J. Li X. Li J. Zheng T. Xie L. (2023). Distinct signatures of gut microbiota and metabolites in different types of diabetes: a population-based cross-sectional study. EClinicalMedicine 62, 102132. 10.1016/j.eclinm.2023.102132 37593224 PMC10430172

[B70] Huang Y. Zhou M. Sun H. Wang Y. (2011). Branched-chain amino acid metabolism in heart disease: an epiphenomenon or a real culprit? Cardiovasc. Res. 90 (2), 220–223. 10.1093/cvr/cvr070 21502372 PMC3078803

[B71] Huang Y. Liu Y. Li L. Su B. Yang L. Fan W. (2014). Involvement of inflammation-related miR-155 and miR-146a in diabetic nephropathy: implications for glomerular endothelial injury. BMC Nephrol. 15, 142. 10.1186/1471-2369-15-142 25182190 PMC4236663

[B72] Huang L. M. Hu Q. Huang X. Qian Y. Lai X. H. (2020). Preconditioning rats with three lipid emulsions prior to acute lung injury affects cytokine production and cell apoptosis in the lung and liver. Lipids health Dis. 19 (1), 19. 10.1186/s12944-019-1137-x 32024527 PMC7003422

[B73] Hughes M. J. McGettrick H. M. Sapey E. (2020). Shared mechanisms of multimorbidity in COPD, atherosclerosis and type-2 diabetes: the neutrophil as a potential inflammatory target. Eur. Respir. Rev. official J. Eur. Respir. Soc. 29 (155), 190102. 10.1183/16000617.0102-2019 32198215 PMC9488696

[B74] Jeyanathan M. Vaseghi-Shanjani M. Afkhami S. Grondin J. A. Kang A. D'Agostino M. R. (2022). Parenteral BCG vaccine induces lung-resident memory macrophages and trained immunity *via* the gut-lung axis. Nat. Immunol. 23 (12), 1687–1702. 10.1038/s41590-022-01354-4 36456739 PMC9747617

[B75] Jiao Y. Zhang T. Liu M. Zhou L. Qi M. Xie X. (2023). Exosomal PGE2 from M2 macrophages inhibits neutrophil recruitment and NET formation through lipid mediator class switching in sepsis. J. Biomed. Sci. 30 (1), 62. 10.1186/s12929-023-00957-9 37533081 PMC10394797

[B76] Jin Y. Dong H. Xia L. Yang Y. Zhu Y. Shen Y. (2019). The diversity of gut Microbiome is associated with favorable responses to anti-programmed death 1 immunotherapy in Chinese patients with NSCLC. J. Thorac. Oncol. official Publ. Int. Assoc. Study Lung Cancer 14 (8), 1378–1389. 10.1016/j.jtho.2019.04.007 31026576

[B77] Jin S. Ding X. Yang C. Li W. Deng M. Liao H. (2021). Mechanical ventilation exacerbates poly (I:C) induced acute lung injury: central role for Caspase-11 and gut-lung axis. Front. Immunol. 12, 693874. 10.3389/fimmu.2021.693874 34349759 PMC8327178

[B78] Jin Y. Wang Y. Feng M. Ni X. Qiang L. Xue J. (2024). Sphingosine-1-phosphate alleviates Sjögren's syndrome-like symptoms *via* inducing autophagy and regulating status of Treg cells in NOD mice. Int. Immunopharmacol. 143 (Pt 3), 113514. 10.1016/j.intimp.2024.113514 39510034

[B79] Jones J. H. Minshall R. D. (2020). Lung Endothelial Transcytosis. Compr. Physiol. 10 (2), 491–508. 10.1002/cphy.c190012 32163197 PMC9819860

[B80] Jung S. J. Hadinoto K. Park J. W. (2023). Mechanical properties of 3-Hydroxybutyric acid-induced vesicles. Mol. Basel, Switz. 28 (6), 2742. 10.3390/molecules28062742 36985713 PMC10051961

[B81] Karcz T. P. Whitehead G. S. Nakano K. Nakano H. Grimm S. A. Williams J. G. (2021). UDP-glucose and P2Y14 receptor amplify allergen-induced airway eosinophilia. J. Clin. investigation 131 (7), e140709. 10.1172/JCI140709 33792561 PMC8011887

[B82] Kai Y. Matsuda M. Suzuki K. Kasamatsu T. Kajita A. Uno K. (2022). Tocilizumab and baricitinib for recovery from acute exacerbation of combined pulmonary fibrosis and emphysema secondary to COVID-19 infection: a case report. Cureus 14 (3), e23411. 10.7759/cureus.23411 35481309 PMC9033509

[B83] Keestra-Gounder A. M. Byndloss M. X. Seyffert N. Young B. M. Chávez-Arroyo A. Tsai A. Y. (2016). NOD1 and NOD2 signalling links ER stress with inflammation. Nature 532 (7599), 394–397. 10.1038/nature17631 27007849 PMC4869892

[B84] Khalid M. Petroianu G. Adem A. (2022). Advanced glycation end products and diabetes mellitus: mechanisms and perspectives. Biomolecules 12 (4), 542. 10.3390/biom12040542 35454131 PMC9030615

[B85] Khanna D. Lin C. J. F. Furst D. E. Wagner B. Zucchetto M. Raghu G. (2022). Long-Term safety and efficacy of Tocilizumab in early systemic sclerosis-interstitial lung disease: Open-Label extension of a phase 3 randomized controlled trial. Am. J. Respir. Crit. care Med. 205 (6), 674–684. 10.1164/rccm.202103-0714OC 34851799

[B86] Khateeb J. Fuchs E. Khamaisi M. (2019). Diabetes and lung disease: a neglected relationship. Rev. Diabet. Stud. RDS 15, 1–15. 10.1900/RDS.2019.15.1 30489598 PMC6760893

[B87] Kheirollahi V. Wasnick R. M. Biasin V. Vazquez-Armendariz A. I. Chu X. Moiseenko A. (2019). Metformin induces lipogenic differentiation in myofibroblasts to reverse lung fibrosis. Nat. Commun. 10 (1), 2987. 10.1038/s41467-019-10839-0 31278260 PMC6611870

[B88] Kim W. Egan J. M. (2008). The role of incretins in glucose homeostasis and diabetes treatment. Pharmacol. Rev. 60 (4), 470–512. 10.1124/pr.108.000604 19074620 PMC2696340

[B89] Kim K. H. Lee M. S. (2014). Autophagy--a key player in cellular and body metabolism. Nat. Rev. Endocrinol. 10 (6), 322–337. 10.1038/nrendo.2014.35 24663220

[B90] Koren O. Konnikova L. Brodin P. Mysorekar I. U. Collado M. C. (2024). The maternal gut microbiome in pregnancy: implications for the developing immune system. Nat. Rev. Gastroenterology and hepatology 21 (1), 35–45. 10.1038/s41575-023-00864-2 38097774 PMC12635954

[B91] Kottmann R. M. Kulkarni A. A. Smolnycki K. A. Lyda E. Dahanayake T. Salibi R. (2012). Lactic acid is elevated in idiopathic pulmonary fibrosis and induces myofibroblast differentiation *via* pH-dependent activation of transforming growth factor-β. Am. J. Respir. Crit. care Med. 186 (8), 740–751. 10.1164/rccm.201201-0084OC 22923663 PMC3480515

[B92] Koukourakis M. I. Kalamida D. Mitrakas A. G. Liousia M. Pouliliou S. Sivridis E. (2017). Metabolic cooperation between co-cultured lung cancer cells and lung fibroblasts. Laboratory investigation; a J. Tech. methods pathology 97 (11), 1321–1331. 10.1038/labinvest.2017.79 28846077

[B93] Kozieł K. Urbanska E. M. (2023). Kynurenine pathway in diabetes mellitus-novel pharmacological target? Cells 12 (3), 460. 10.3390/cells12030460 36766803 PMC9913876

[B94] Kuda O. Jenkins C. M. Skinner J. R. Moon S. H. Su X. Gross R. W. (2011). CD36 protein is involved in store-operated calcium flux, phospholipase A2 activation, and production of prostaglandin E2. J. Biol. Chem. 286 (20), 17785–17795. 10.1074/jbc.M111.232975 21454644 PMC3093854

[B95] Kukreja A. Cost G. Marker J. Zhang C. Sun Z. Lin-Su K. (2002). Multiple immuno-regulatory defects in type-1 diabetes. J. Clin. investigation 109 (1), 131–140. 10.1172/JCI13605 11781358 PMC150819

[B96] Kwon S. Crowley G. Caraher E. J. Haider S. H. Lam R. Veerappan A. (2019). Validation of predictive metabolic syndrome biomarkers of world Trade Center lung injury: a 16-Year longitudinal Study. Chest 156 (3), 486–496. 10.1016/j.chest.2019.02.019 30836056 PMC6717118

[B97] Lai H. C. Lin T. L. Chen T. W. Kuo Y. L. Chang C. J. Wu T. R. (2022). Gut microbiota modulates COPD pathogenesis: role of anti-inflammatory Parabacteroides goldsteinii lipopolysaccharide. Gut 71 (2), 309–321. 10.1136/gutjnl-2020-322599 33687943

[B98] Lange P. Groth S. Kastrup J. Mortensen J. Appleyard M. Nyboe J. (1989). Diabetes mellitus, plasma glucose and lung function in a cross-sectional population study. Eur. Respir. J. 2 (1), 14–19. 10.1183/09031936.93.02010014 2651148

[B99] Lee B. Man K. K. C. Wong E. Tan T. Sheikh A. Bloom C. I. (2025). Antidiabetic medication and asthma attacks. JAMA Intern. Med. 185 (1), 16–25. 10.1001/jamainternmed.2024.5982 39556360 PMC11574725

[B100] Legchenko E. Chouvarine P. Borchert P. Fernandez-Gonzalez A. Snay E. Meier M. (2018). PPARγ agonist pioglitazone reverses pulmonary hypertension and prevents right heart failure *via* fatty acid oxidation. Sci. Transl. Med. 10 (438), eaao0303. 10.1126/scitranslmed.aao0303 29695452

[B101] Li S. Yang H. (2019). Relationship between advanced glycation end products and gestational diabetes mellitus. J. maternal-fetal and neonatal Med. official J. Eur. Assoc. Perinat. Med. Fed. Asia Ocean. Perinat. Soc. Int. Soc. Perinat. Obstet 32 (17), 2783–2789. 10.1080/14767058.2018.1449201 29560756

[B102] Li K. Li M. Li W. Yu H. Sun X. Zhang Q. (2019). Airway epithelial regeneration requires autophagy and glucose metabolism. Cell. death and Dis. 10 (12), 875. 10.1038/s41419-019-2111-2 31748541 PMC6868131

[B103] Li M. Zhang C. S. Feng J. W. Wei X. Zhang C. Xie C. (2021). Aldolase is a sensor for both low and high glucose, linking to AMPK and mTORC1. Cell. Res. 31 (4), 478–481. 10.1038/s41422-020-00456-8 33349646 PMC8115481

[B104] Li C. Gao P. Zhuang F. Wang T. Wang Z. Wu G. (2024). Inhibition of ALOX12-12-HETE alleviates lung ischemia-reperfusion injury by reducing endothelial ferroptosis-mediated neutrophil extracellular trap Formation, 7:0473. 10.34133/research.0473 39268501 PMC11391482

[B105] Lin F. Song C. Zeng Y. Li Y. Li H. Liu B. (2020). Canagliflozin alleviates LPS-induced acute lung injury by modulating alveolar macrophage polarization. Int. Immunopharmacol. 88, 106969. 10.1016/j.intimp.2020.106969 33182027

[B106] Ling C. Bacos K. Rönn T. (2022). Epigenetics of type 2 diabetes mellitus and weight change - a tool for precision medicine? Nat. Rev. Endocrinol. 18 (7), 433–448. 10.1038/s41574-022-00671-w 35513492

[B107] Ling K. M. Stick S. M. Kicic A. (2023). Pulmonary bacteriophage and cystic fibrosis airway mucus: friends or foes? Front. Med. 10, 1088494. 10.3389/fmed.2023.1088494 37265479 PMC10230084

[B108] Liu G. Summer R. (2019). Cellular metabolism in lung health and disease. Annu. Rev. physiology 81, 403–428. 10.1146/annurev-physiol-020518-114640 30485759 PMC6853603

[B109] Liu S. Li L. Lou P. Zhao M. Wang Y. Tang M. (2021a). Elevated branched-chain α-keto acids exacerbate macrophage oxidative stress and chronic inflammatory damage in type 2 diabetes mellitus. Free Radic. Biol. and Med. 175, 141–154. 10.1016/j.freeradbiomed.2021.08.240 34474107

[B110] Liu Q. Tian X. Maruyama D. Arjomandi M. Prakash A. (2021b). Lung immune tone *via* gut-lung axis: gut-derived LPS and short-chain fatty acids' immunometabolic regulation of lung IL-1β, FFAR2, and FFAR3 expression. Am. J. physiology Lung Cell. Mol. physiology 321 (1), L65–l78. 10.1152/ajplung.00421.2020 33851870 PMC8321849

[B111] Liu N. Bai L. Lu Z. Gu R. Zhao D. Yan F. (2022). TRPV4 contributes to ER stress and inflammation: implications for Parkinson's disease. J. neuroinflammation 19 (1), 26. 10.1186/s12974-022-02382-5 35093118 PMC8800324

[B112] Lo J. Clare-Salzler M. J. (2006). Dendritic cell subsets and type I diabetes: focus upon DC-based therapy. Autoimmun. Rev. 5 (6), 419–423. 10.1016/j.autrev.2005.12.001 16890897

[B113] Lynch C. J. Adams S. H. (2014). Branched-chain amino acids in metabolic signalling and insulin resistance. Nat. Rev. Endocrinol. 10 (12), 723–736. 10.1038/nrendo.2014.171 25287287 PMC4424797

[B114] Ma R. Ji T. Zhang H. Dong W. Chen X. Xu P. (2018). A Pck1-directed glycogen metabolic program regulates formation and maintenance of memory CD8(+) T cells. Nat. Cell. Biol. 20 (1), 21–27. 10.1038/s41556-017-0002-2 29230018

[B115] Ma J. Wei K. Liu J. Tang K. Zhang H. Zhu L. (2020). Glycogen metabolism regulates macrophage-mediated acute inflammatory responses. Nat. Commun. 11 (1), 1769. 10.1038/s41467-020-15636-8 32286295 PMC7156451

[B116] Ma X. Mei S. Wuyun Q. Zhou L. Cai Z. Ding H. (2024). Super-enhancer-driven LncRNA PPARα-seRNA exacerbates glucolipid metabolism and diabetic cardiomyopathy *via* recruiting KDM4B. Mol. Metab. 86, 101978. 10.1016/j.molmet.2024.101978 38950776 PMC11277359

[B117] Ma S. Wang M. Wu Y. Meng D. Zhang B. Zhu H. (2025). Discovery of a series of novel 3-sulfonamido benzoic acid derivatives as promising P2Y(14)R antagonists for acute lung injury. Eur. J. Med. Chem. 290, 117588. 10.1016/j.ejmech.2025.117588 40179611

[B118] Magnotti L. J. Upperman J. S. Xu D. Z. Lu Q. Deitch E. A. (1998). Gut-derived mesenteric lymph but not portal blood increases endothelial cell permeability and promotes lung injury after hemorrhagic shock. Ann. Surg. 228 (4), 518–527. 10.1097/00000658-199810000-00008 9790341 PMC1191527

[B119] Majer M. Mott D. M. Mochizuki H. Rowles J. C. Pedersen O. Knowler W. C. (1996). Association of the glycogen synthase locus on 19q13 with NIDDM in Pima Indians. Diabetologia 39 (3), 314–321. 10.1007/BF00418347 8721777

[B120] Mao L. Wang L. Lyu Y. Zhuang Q. Li Z. Zhang J. (2024). Branch chain amino acid metabolism promotes brain metastasis of NSCLC through EMT occurrence by regulating ALKBH5 activity. Int. J. Biol. Sci. 20 (9), 3285–3301. 10.7150/ijbs.85672 38993559 PMC11234221

[B121] Martín-Vázquez E. Cobo-Vuilleumier N. López-Noriega L. Lorenzo P. I. Gauthier B. R. (2023). The PTGS2/COX2-PGE(2) signaling cascade in inflammation: pro or anti? A case study with type 1 diabetes mellitus. Int. J. Biol. Sci. 19 (13), 4157–4165. 10.7150/ijbs.86492 37705740 PMC10496497

[B122] Maschalidi S. Mehrotra P. Keçeli B. N. De Cleene H. K. L. Lecomte K. Van der Cruyssen R. (2022). Targeting SLC7A11 improves efferocytosis by dendritic cells and wound healing in diabetes. Nature 606 (7915), 776–784. 10.1038/s41586-022-04754-6 35614212

[B123] Mayers J. R. Torrence M. E. Danai L. V. Papagiannakopoulos T. Davidson S. M. Bauer M. R. (2016). Tissue of origin dictates branched-chain amino acid metabolism in mutant Kras-driven cancers. Sci. (New York, NY) 353 (6304), 1161–1165. 10.1126/science.aaf5171 27609895 PMC5245791

[B124] Mendez-Enriquez E. Alvarado-Vazquez P. A. Abma W. Simonson O. E. Rodin S. Feyerabend T. B. (2021). Mast cell-derived serotonin enhances methacholine-induced airway hyperresponsiveness in house dust mite-induced experimental asthma. Allergy 76 (7), 2057–2069. 10.1111/all.14748 33486786

[B125] Mishra S. P. Wang B. Jain S. Ding J. Rejeski J. Furdui C. M. (2023). A mechanism by which gut microbiota elevates permeability and inflammation in obese/diabetic mice and human gut. Gut 72 (10), 1848–1865. 10.1136/gutjnl-2022-327365 36948576 PMC10512000

[B126] Molinaro A. Bel Lassen P. Henricsson M. Wu H. Adriouch S. Belda E. (2020). Imidazole propionate is increased in diabetes and associated with dietary patterns and altered microbial ecology. Nat. Commun. 11 (1), 5881. 10.1038/s41467-020-19589-w 33208748 PMC7676231

[B127] Montuschi P. Kharitonov S. A. Ciabattoni G. Barnes P. J. (2003). Exhaled leukotrienes and prostaglandins in COPD. Thorax 58 (7), 585–588. 10.1136/thorax.58.7.585 12832671 PMC1746732

[B128] Mousavinasab F. Karimi R. Taheri S. Ahmadvand F. Sanaaee S. Najafi S. (2023). Microbiome modulation in inflammatory diseases: progress to microbiome genetic engineering. Cancer Cell. Int. 23 (1), 271. 10.1186/s12935-023-03095-2 37951913 PMC10640760

[B129] Myerburg M. M. King J. D. Jr. Oyster N. M. Fitch A. C. Magill A. Baty C. J. (2010). AMPK agonists ameliorate sodium and fluid transport and inflammation in cystic fibrosis airway epithelial cells. Am. J. Respir. Cell. Mol. Biol. 42 (6), 676–684. 10.1165/2009-0147OC 19617399 PMC2891496

[B130] Nagata N. Takeuchi T. Masuoka H. Aoki R. Ishikane M. Iwamoto N. (2023). Human gut Microbiota and its metabolites impact immune responses in COVID-19 and its complications. Gastroenterology 164 (2), 272–288. 10.1053/j.gastro.2022.09.024 36155191 PMC9499989

[B131] Natalini J. G. Singh S. Segal L. N. (2023). The dynamic lung microbiome in health and disease. Nat. Rev. Microbiol. 21 (4), 222–235. 10.1038/s41579-022-00821-x 36385637 PMC9668228

[B132] Naveen Kumar R. Surekha M. V. Gowthami S. D. G. Aditi A. K. Satyavani M. Satyanarayana K. (2025). Toxicological evaluation of Salmonella phage NINP13076 in BALB/c mice: histopathological studies. Microb. Pathog. 198, 107146. 10.1016/j.micpath.2024.107146 39586340

[B133] Newgard C. B. An J. Bain J. R. Muehlbauer M. J. Stevens R. D. Lien L. F. (2009). A branched-chain amino acid-related metabolic signature that differentiates obese and lean humans and contributes to insulin resistance. Cell. metab. 9 (4), 311–326. 10.1016/j.cmet.2009.02.002 19356713 PMC3640280

[B134] Ng S. C. Xu Z. Mak J. W. Y. Yang K. Liu Q. Zuo T. (2022). Microbiota engraftment after faecal microbiota transplantation in obese subjects with type 2 diabetes: a 24-week, double-blind, randomised controlled trial. Gut 71 (4), 716–723. 10.1136/gutjnl-2020-323617 33785557

[B135] Nguyen S. Baker K. Padman B. S. Patwa R. Dunstan R. A. Weston T. A. (2017). Bacteriophage transcytosis provides a mechanism to cross epithelial cell layers. mBio 8 (6), e01874-17. 10.1128/mBio.01874-17 29162715 PMC5698557

[B136] Nishijima S. Nagata N. Kiguchi Y. Kojima Y. Miyoshi-Akiyama T. Kimura M. (2022). Extensive gut virome variation and its associations with host and environmental factors in a population-level cohort. Nat. Commun. 13 (1), 5252. 10.1038/s41467-022-32832-w 36068216 PMC9448778

[B137] Nobs S. P. Kopf M. (2018). PPAR-γ in innate and adaptive lung immunity. J. Leukoc. Biol. 104 (4), 737–741. 10.1002/JLB.3MR0118-034R 29768688

[B138] Nobs S. P. Kolodziejczyk A. A. Adler L. Horesh N. Botscharnikow C. Herzog E. (2023). Lung dendritic-cell metabolism underlies susceptibility to viral infection in diabetes. Nature 624 (7992), 645–652. 10.1038/s41586-023-06803-0 38093014 PMC10733144

[B139] O'Keefe J. H. Weidling R. O'Keefe E. L. Franco W. G. (2023). SGLT inhibitors for improving Healthspan and lifespan. Prog. Cardiovasc. Dis. 81, 2–9. 10.1016/j.pcad.2023.10.003 37852518 PMC10831928

[B140] Okla M. Kim J. Koehler K. Chung S. (2017). Dietary factors promoting brown and beige fat development and thermogenesis. Adv. Nutr. (Bethesda, Md) 8 (3), 473–483. 10.3945/an.116.014332 28507012 PMC5421122

[B141] Otto M. Bucher C. Liu W. Müller M. Schmidt T. Kardell M. (2020). 12(S)-HETE mediates diabetes-induced endothelial dysfunction by activating intracellular endothelial cell TRPV1. J. Clin. investigation 130 (9), 4999–5010. 10.1172/JCI136621 32584793 PMC7456227

[B142] Özçam M. Lynch S. V. (2024). The gut-airway microbiome axis in health and respiratory diseases. Nat. Rev. Microbiol. 22 (8), 492–506. 10.1038/s41579-024-01048-8 38778224 PMC12051635

[B143] Packer M. (2020). Role of impaired nutrient and oxygen deprivation signaling and deficient autophagic flux in diabetic CKD development: implications for understanding the effects of sodium-glucose cotransporter 2-Inhibitors. J. Am. Soc. Nephrol. JASN 31 (5), 907–919. 10.1681/ASN.2020010010 32276962 PMC7217421

[B144] Patkee W. R. Carr G. Baker E. H. Baines D. L. Garnett J. P. (2016). Metformin prevents the effects of Pseudomonas aeruginosa on airway epithelial tight junctions and restricts hyperglycaemia-induced bacterial growth. J. Cell. Mol. Med. 20 (4), 758–764. 10.1111/jcmm.12784 26837005 PMC4864950

[B145] Pernet E. Sun S. Sarden N. Gona S. Nguyen A. Khan N. (2023). Neonatal imprinting of alveolar macrophages *via* neutrophil-derived 12-HETE. Nature 614 (7948), 530–538. 10.1038/s41586-022-05660-7 36599368 PMC9945843

[B146] Petrus P. Lecoutre S. Dollet L. Wiel C. Sulen A. Gao H. (2020). Glutamine links obesity to inflammation in Human white adipose tissue. Cell. metab. 31 (2), 375–390. 10.1016/j.cmet.2019.11.019 31866443

[B147] Poltorak A. He X. Smirnova I. Liu M. Y. Van Huffel C. Du X. (1998). Defective LPS signaling in C3H/HeJ and C57BL/10ScCr mice: mutations in Tlr4 gene. Sci. (New York, NY) 282 (5396), 2085–2088. 10.1126/science.282.5396.2085 9851930

[B148] Popov D. Simionescu M. (1997). Alterations of lung structure in experimental diabetes, and diabetes associated with hyperlipidaemia in hamsters. Eur. Respir. J. 10 (8), 1850–1858. 10.1183/09031936.97.10081850 9272930

[B149] Prattichizzo F. de Candia P. Ceriello A. (2021). Diabetes and kidney disease: emphasis on treatment with SGLT-2 inhibitors and GLP-1 receptor agonists. Metabolism Clin. Exp. 120, 154799. 10.1016/j.metabol.2021.154799 34029597

[B150] Prieto I. Kavanagh M. Jimenez-Castilla L. Pardines M. Lazaro I. Herrero Del Real I. (2023). A mutual regulatory loop between miR-155 and SOCS1 influences renal inflammation and diabetic kidney disease. Mol. Ther. Nucleic acids 34, 102041. 10.1016/j.omtn.2023.102041 37842165 PMC10571033

[B151] Qian X. Zhang H. Y. Li Q. L. Ma G. J. Chen Z. Ji X. M. (2022). Integrated microbiome, metabolome, and proteome analysis identifies a novel interplay among commensal bacteria, metabolites and candidate targets in non-small cell lung cancer. Clin. Transl. Med. 12 (6), e947. 10.1002/ctm2.947 35735103 PMC9218934

[B152] Rajesh R. Atallah R. Bärnthaler T. (2023). Dysregulation of metabolic pathways in pulmonary fibrosis. Pharmacol. and Ther. 246, 108436. 10.1016/j.pharmthera.2023.108436 37150402

[B153] Retamal J. S. Grace M. S. Dill L. K. Ramirez-Garcia P. Peng S. Gondin A. B. (2021). Serotonin-induced vascular permeability is mediated by transient receptor potential vanilloid 4 in the airways and upper gastrointestinal tract of mice. Laboratory investigation; a J. Tech. methods pathology 101 (7), 851–864. 10.1038/s41374-021-00593-7 33859334 PMC8047529

[B154] Roberts T. J. Burns A. T. MacIsaac R. J. MacIsaac A. I. Prior D. L. La Gerche A. (2018). Diagnosis and significance of pulmonary microvascular disease in diabetes. Diabetes care 41 (4), 854–861. 10.2337/dc17-1904 29351959

[B155] Rogliani P. Calzetta L. Capuani B. Facciolo F. Cazzola M. Lauro D. (2016). Glucagon-Like peptide 1 receptor: a novel pharmacological target for treating human bronchial hyperresponsiveness. Am. J. Respir. Cell. Mol. Biol. 55 (6), 804–814. 10.1165/rcmb.2015-0311OC 27447052

[B156] Rohm T. V. Keller L. Bosch A. J. T. AlAsfoor S. Baumann Z. Thomas A. (2022). Targeting colonic macrophages improves glycemic control in high-fat diet-induced obesity. Commun. Biol. 5 (1), 370. 10.1038/s42003-022-03305-z 35440795 PMC9018739

[B157] Romero F. Shah D. Duong M. Penn R. B. Fessler M. B. Madenspacher J. (2015). A pneumocyte-macrophage paracrine lipid axis drives the lung toward fibrosis. Am. J. Respir. Cell. Mol. Biol. 53 (1), 74–86. 10.1165/rcmb.2014-0343OC 25409201 PMC4566113

[B158] Ruderman N. B. Carling D. Prentki M. Cacicedo J. M. (2013). AMPK, insulin resistance, and the metabolic syndrome. J. Clin. investigation 123 (7), 2764–2772. 10.1172/JCI67227 23863634 PMC3696539

[B159] Saad M. J. Santos A. Prada P. O. (2016). Linking gut microbiota and inflammation to obesity and insulin resistance. Physiol. (Bethesda, Md) 31 (4), 283–293. 10.1152/physiol.00041.2015 27252163

[B160] Sacks D. B. Arnold M. Bakris G. L. Bruns D. E. Horvath A. R. Lernmark Å. (2023). Guidelines and recommendations for laboratory analysis in the diagnosis and management of diabetes mellitus. Diabetes care 46 (10), e151–e199. 10.2337/dci23-0036 37471273 PMC10516260

[B161] Sas K. M. Kayampilly P. Byun J. Nair V. Hinder L. M. Hur J. (2016). Tissue-specific metabolic reprogramming drives nutrient flux in diabetic complications. JCI insight 1 (15), e86976. 10.1172/jci.insight.86976 27699244 PMC5033761

[B162] Schipke J. Jütte D. Brandenberger C. Autilio C. Perez-Gil J. Bernhard W. (2021). Dietary carbohydrates and fat induce distinct surfactant alterations in mice. Am. J. Respir. Cell. Mol. Biol. 64 (3), 379–390. 10.1165/rcmb.2020-0335OC 33351709

[B163] Schoeler M. Caesar R. (2019). Dietary lipids, gut microbiota and lipid metabolism. Rev. Endocr. and metabolic Disord. 20 (4), 461–472. 10.1007/s11154-019-09512-0 31707624 PMC6938793

[B164] Shahwan M. Alhumaydhi F. Ashraf G. M. Hasan P. M. Z. Shamsi A. (2022). Role of polyphenols in combating Type 2 Diabetes and insulin resistance. Int. J. Biol. Macromol. 206, 567–579. 10.1016/j.ijbiomac.2022.03.004 35247420

[B165] Shakour N. Karami S. Iranshahi M. Butler A. E. Sahebkar A. (2024). Antifibrotic effects of sodium-glucose cotransporter-2 inhibitors: a comprehensive review. Diabetes and metabolic syndrome 18 (1), 102934. 10.1016/j.dsx.2023.102934 38154403

[B166] Shi H. Yuan X. Yang X. Huang R. Fan W. Liu G. (2024). A novel diabetic foot ulcer diagnostic model: identification and analysis of genes related to glutamine metabolism and immune infiltration. BMC genomics 25 (1), 125. 10.1186/s12864-024-10038-2 38287255 PMC10826017

[B167] Shieh S. H. Probst J. C. Sung F. C. Tsai W. C. Li Y. S. Chen C. Y. (2012). Decreased survival among lung cancer patients with co-morbid tuberculosis and diabetes. BMC cancer 12, 174. 10.1186/1471-2407-12-174 22578056 PMC3408323

[B168] Shkoporov A. N. Stockdale S. R. Lavelle A. Kondova I. Heuston C. Upadrasta A. (2022). Viral biogeography of the mammalian gut and parenchymal organs. Nat. Microbiol. 7 (8), 1301–1311. 10.1038/s41564-022-01178-w 35918425 PMC7614033

[B169] Singh A. K. Gillies C. L. Singh R. Singh A. Chudasama Y. Coles B. (2020). Prevalence of co-morbidities and their association with mortality in patients with COVID-19: a systematic review and meta-analysis. Diabetes, Obes. and metabolism 22 (10), 1915–1924. 10.1111/dom.14124 32573903 PMC7361304

[B170] Slatore C. G. Bryson C. L. Au D. H. (2009). The association of inhaled corticosteroid use with serum glucose concentration in a large cohort. Am. J. Med. 122 (5), 472–478. 10.1016/j.amjmed.2008.09.048 19375557

[B171] Smith P. M. Howitt M. R. Panikov N. Michaud M. Gallini C. A. Bohlooly Y. M. (2013). The microbial metabolites, short-chain fatty acids, regulate colonic Treg cell homeostasis. Sci. (New York, NY) 341 (6145), 569–573. 10.1126/science.1241165 23828891 PMC3807819

[B172] Sodhi C. P. Jia H. Yamaguchi Y. Lu P. Good M. Egan C. (2015). Intestinal epithelial TLR-4 activation is required for the development of Acute lung injury after Trauma/Hemorrhagic shock *via* the release of HMGB1 from the gut. J. Immunol. Baltim. Md 1950 194 (10), 4931–4939. 10.4049/jimmunol.1402490 25862813 PMC4417407

[B173] Stapleton D. Mitchelhill K. I. Gao G. Widmer J. Michell B. J. Teh T. (1996). Mammalian AMP-activated protein kinase subfamily. J. Biol. Chem. 271 (2), 611–614. 10.1074/jbc.271.2.611 8557660

[B174] Stevens J. Steinmeyer S. Bonfield M. Peterson L. Wang T. Gray J. (2022). The balance between protective and pathogenic immune responses to pneumonia in the neonatal lung is enforced by gut microbiota. Sci. Transl. Med. 14 (649), eabl3981. 10.1126/scitranslmed.abl3981 35704600 PMC10032669

[B175] Tacke F. Weiskirchen R. (2018). An update on the recent advances in antifibrotic therapy. Expert Rev. gastroenterology and hepatology 12 (11), 1143–1152. 10.1080/17474124.2018.1530110 30261763

[B176] Tanaka T. Narazaki M. Kishimoto T. (2018). Interleukin (IL-6) immunotherapy. Cold Spring Harb. Perspect. Biol. 10 (8), a028456. 10.1101/cshperspect.a028456 28778870 PMC6071487

[B177] Tanase D. M. Gosav E. M. Neculae E. Costea C. F. Ciocoiu M. Hurjui L. L. (2020). Role of Gut Microbiota on onset and progression of microvascular complications of type 2 diabetes (T2DM). Nutrients 12 (12), 3719. 10.3390/nu12123719 33276482 PMC7760723

[B178] Tangedal S. Nielsen R. Aanerud M. Drengenes C. Husebø G. R. Lehmann S. (2024). Lower airway microbiota in COPD and healthy controls. Thorax 79, 219–226. 10.1136/thorax-2023-220455 38331579

[B179] Thannickal V. J. Fanburg B. L. (2000). Reactive oxygen species in cell signaling. Am. J. physiology Lung Cell. Mol. physiology 279 (6), L1005–L1028. 10.1152/ajplung.2000.279.6.L1005 11076791

[B180] Thomas T. Stefanoni D. Reisz J. A. Nemkov T. Bertolone L. Francis R. O. (2020). COVID-19 infection alters kynurenine and fatty acid metabolism, correlating with IL-6 levels and renal status. JCI insight 5 (14), e140327. 10.1172/jci.insight.140327 32559180 PMC7453907

[B181] Tiengo A. Fadini G. P. Avogaro A. (2008). The metabolic syndrome, diabetes and lung dysfunction. Diabetes and metabolism 34 (5), 447–454. 10.1016/j.diabet.2008.08.001 18829364

[B182] Toki S. Newcomb D. C. Printz R. L. Cahill K. N. Boyd K. L. Niswender K. D. (2021). Glucagon-like peptide-1 receptor agonist inhibits aeroallergen-induced activation of ILC2 and neutrophilic airway inflammation in obese mice. Allergy 76 (11), 3433–3445. 10.1111/all.14879 33955007 PMC8597133

[B183] Uno K. Yamada T. Ishigaki Y. Imai J. Hasegawa Y. Sawada S. (2015). A hepatic amino acid/mTOR/S6K-dependent signalling pathway modulates systemic lipid metabolism *via* neuronal signals. Nat. Commun. 6, 7940. 10.1038/ncomms8940 26268630 PMC4557134

[B184] van Hoek M. J. Merks R. M. (2012). Redox balance is key to explaining full vs. partial switching to low-yield metabolism. BMC Syst. Biol. 6, 22. 10.1186/1752-0509-6-22 22443685 PMC3384451

[B185] Velmurugan G. Ramprasath T. Gilles M. Swaminathan K. Ramasamy S. (2017). Gut Microbiota, endocrine-disrupting chemicals, and the diabetes epidemic. Trends Endocrinol. metabolism TEM 28 (8), 612–625. 10.1016/j.tem.2017.05.001 28571659

[B186] Verkerke A. R. P. Wang D. Yoshida N. Taxin Z. H. Shi X. Zheng S. (2024). BCAA-nitrogen flux in brown fat controls metabolic health independent of thermogenesis. Cell. 187 (10), 2359–2374.e18. 10.1016/j.cell.2024.03.030 38653240 PMC11145561

[B187] Wang T. J. Larson M. G. Vasan R. S. Cheng S. Rhee E. P. McCabe E. (2011). Metabolite profiles and the risk of developing diabetes. Nat. Med. 17 (4), 448–453. 10.1038/nm.2307 21423183 PMC3126616

[B188] Wang Y. Xiao J. Jiang W. Zuo D. Wang X. Jin Y. (2021a). BCKDK alters the metabolism of non-small cell lung cancer. Transl. lung cancer Res. 10 (12), 4459–4476. 10.21037/tlcr-21-885 35070754 PMC8743533

[B189] Wang C. Xie K. Li K. Lin S. Xu F. (2021b). Potential therapeutic effects of interleukin-35 on the differentiation of naïve T cells into Helios+Foxp3+ Tregs in clinical and experimental acute respiratory distress syndrome. Mol. Immunol. 132, 236–249. 10.1016/j.molimm.2021.01.009 33494935 PMC8058740

[B190] Wang L. Wang J. Ren G. Sun S. Nishikawa K. Yu J. (2023a). Ameliorative effects of the Coptis inflorescence extract against lung injury in diabetic mice by regulating AMPK/NEU1 signaling. Phytomedicine Int. J. phytotherapy Phytopharm. 118, 154963. 10.1016/j.phymed.2023.154963 37516057

[B191] Wang Y. Deng F. Zhong X. Du Y. Fan X. Su H. (2023b). Dulaglutide provides protection against sepsis-induced lung injury in mice by inhibiting inflammation and apoptosis. Eur. J. Pharmacol. 949, 175730. 10.1016/j.ejphar.2023.175730 37062504

[B192] Webber T. Ronacher K. Conradie-Smit M. Kleynhans L. (2022). Interplay between the immune and endocrine systems in the lung: implications for TB susceptibility. Front. Immunol. 13, 829355. 10.3389/fimmu.2022.829355 35273609 PMC8901994

[B193] Willart M. A. van Nimwegen M. Grefhorst A. Hammad H. Moons L. Hoogsteden H. C. (2012). Ursodeoxycholic acid suppresses eosinophilic airway inflammation by inhibiting the function of dendritic cells through the nuclear farnesoid X receptor. Allergy 67 (12), 1501–1510. 10.1111/all.12019 23004356

[B194] Wolfe A. L. Zhou Q. Toska E. Galeas J. Ku A. A. Koche R. P. (2021). UDP-glucose pyrophosphorylase 2, a regulator of glycogen synthesis and glycosylation, is critical for pancreatic cancer growth. Proc. Natl. Acad. Sci. U. S. A. 118 (31), e2103592118. 10.1073/pnas.2103592118 34330832 PMC8346792

[B195] Wong C. K. McLean B. A. Baggio L. L. Koehler J. A. Hammoud R. Rittig N. (2024). Central glucagon-like peptide 1 receptor activation inhibits toll-like receptor agonist-induced inflammation. Cell. metab. 36 (1), 130–143.e5. 10.1016/j.cmet.2023.11.009 38113888

[B196] Wu Z. Zhang B. Chen F. Xia R. Zhu D. Chen B. (2022). Fecal microbiota transplantation reverses insulin resistance in type 2 diabetes: a randomized, controlled, prospective study. Front. Cell. Infect. Microbiol. 12, 1089991. 10.3389/fcimb.2022.1089991 36704100 PMC9872724

[B197] Wu J. Gong L. Li Y. Liu T. Sun R. Jia K. (2024). SGK1 aggravates idiopathic pulmonary fibrosis by triggering H3k27ac-mediated macrophage reprogramming and disturbing immune homeostasis. Int. J. Biol. Sci. 20 (3), 968–986. 10.7150/ijbs.90808 38250161 PMC10797695

[B198] Xu W. Janocha A. J. Erzurum S. C. (2021). Metabolism in pulmonary hypertension. Annu. Rev. physiology 83, 551–576. 10.1146/annurev-physiol-031620-123956 33566674 PMC8597719

[B199] Xu R. Wang F. Zhang Z. Zhang Y. Tang Y. Bi J. (2023a). Diabetes-Induced autophagy dysregulation engenders testicular impairment *via* oxidative stress. Oxidative Med. Cell. Longev. 2023, 4365895. 10.1155/2023/4365895 36778206 PMC9918358

[B200] Xu Y. Zhang C. Cai D. Zhu R. Cao Y. (2023b). Exosomal miR-155-5p drives widespread macrophage M1 polarization in hypervirulent Klebsiella pneumoniae-induced acute lung injury *via* the MSK1/p38-MAPK axis. Cell. and Mol. Biol. Lett. 28 (1), 92. 10.1186/s11658-023-00505-1 37953267 PMC10641976

[B201] Xue M. Xiao J. Jiang W. Wang Y. Zuo D. An H. (2023). Loss of BCAA catabolism enhances Rab1A-mTORC1 signaling activity and promotes tumor proliferation in NSCLC. Transl. Oncol. 34, 101696. 10.1016/j.tranon.2023.101696 37216755 PMC10209880

[B202] Yang G. Wei J. Liu P. Zhang Q. Tian Y. Hou G. (2021). Role of the gut microbiota in type 2 diabetes and related diseases. Metabolism Clin. Exp. 117, 154712. 10.1016/j.metabol.2021.154712 33497712

[B203] Yang F. Luo X. Li J. Lei Y. Zeng F. Huang X. (2022). Application of glucagon-like peptide-1 receptor antagonists in fibrotic diseases. Biomed. and Pharmacother. = Biomedecine and Pharmacother. 152, 113236. 10.1016/j.biopha.2022.113236 35691154

[B204] Yang M. Cao Z. Li W. Zhou J. Liu J. Zhong Y. (2024). Maternal glycemia during pregnancy and child lung function: a prospective cohort Study. Diabetes care 47 (11), 1941–1948. 10.2337/dc24-0865 39231019 PMC11502530

[B205] Yao C. C. Sun R. M. Yang Y. Zhou H. Y. Meng Z. W. Chi R. (2023). Accumulation of branched-chain amino acids reprograms glucose metabolism in CD8(+) T cells with enhanced effector function and anti-tumor response. Cell. Rep. 42 (3), 112186. 10.1016/j.celrep.2023.112186 36870057

[B206] Ying W. Riopel M. Bandyopadhyay G. Dong Y. Birmingham A. Seo J. B. (2017). Adipose tissue macrophage-derived exosomal miRNAs can modulate *in vivo* and *in vitro* insulin sensitivity. Cell. 171 (2), 372–384. 10.1016/j.cell.2017.08.035 28942920

[B207] Yoneda T. Yoshikawa M. Tsukaguchi K. Tokuyama T. Fu A. Tomoda K. (1992). Relation of airway obstruction and respiratory muscle weakness to energy metabolism in pulmonary emphysema. Nihon Kyobu Shikkan Gakkai zasshi 30 (9), 1667–1672. 1447842

[B208] Yoo Y. M. Jung E. M. Jeon B. H. Tran D. N. Jeung E. B. (2020). Potassium-dependent sodium/calcium exchanger 3 (Nckx3) depletion leads to abnormal motor function and social behavior in mice. J. physiology Pharmacol. official J. Pol. Physiological Soc. 71 (5). 10.26402/jpp.2020.4.08 33214341

[B209] Yoon H. S. Cho C. H. Yun M. S. Jang S. J. You H. J. Kim J. H. (2021). Akkermansia muciniphila secretes a glucagon-like peptide-1-inducing protein that improves glucose homeostasis and ameliorates metabolic disease in mice. Nat. Microbiol. 6 (5), 563–573. 10.1038/s41564-021-00880-5 33820962

[B210] Yu Z. Ohba M. Nakamura M. Sasano T. Ono M. Sugawara S. (2009). Dynamics of platelet mobilisation into lungs in response to 5-hydroxytryptamine (serotonin) in mice. Thrombosis haemostasis 102 (6), 1251–1258. 10.1160/TH08-06-0406 19967158

[B211] Zangara M. T. Johnston I. Johnson E. E. McDonald C. (2021). Mediators of metabolism: an unconventional role for NOD1 and NOD2. Int. J. Mol. Sci. 22 (3), 1156. 10.3390/ijms22031156 33503814 PMC7866072

[B212] Zhang C. Ma C. Yao H. Zhang L. Yu X. Liu Y. (2018). 12-Lipoxygenase and 12-hydroxyeicosatetraenoic acid regulate hypoxic angiogenesis and survival of pulmonary artery endothelial cells *via* PI3K/Akt pathway. Am. J. physiology Lung Cell. Mol. physiology 314 (4), L606-L616–l616. 10.1152/ajplung.00049.2017 29074487

[B213] Zhang Y. Zhang H. Li S. Huang K. Jiang L. Wang Y. (2022a). Metformin alleviates LPS-Induced acute lung injury by regulating the SIRT1/NF-κB/NLRP3 pathway and inhibiting endothelial cell pyroptosis. Front. Pharmacol. 13, 801337. 10.3389/fphar.2022.801337 35910360 PMC9334876

[B214] Zhang J. Wang H. Chen H. Li H. Xu P. Liu B. (2022b). ATF3 -activated accelerating effect of LINC00941/lncIAPF on fibroblast-to-myofibroblast differentiation by blocking autophagy depending on ELAVL1/HuR in pulmonary fibrosis. Autophagy 18 (11), 2636–2655. 10.1080/15548627.2022.2046448 35427207 PMC9629064

[B215] Zhang Z. Li X. Guo J. He B. Wu L. Yang R. (2023). β-aminoisobutyrics acid, a metabolite of BCAA, activates the AMPK/Nrf-2 pathway to prevent ferroptosis and ameliorates lung ischemia-reperfusion injury. Mol. Med. Camb. Mass 29 (1), 164. 10.1186/s10020-023-00729-z 38049750 PMC10696792

[B216] Zhao W. Zhao B. Meng X. Li B. Wang Y. Yu F. (2024). The regulation of MFG-E8 on the mitophagy in diabetic sarcopenia *via* the HSPA1L-Parkin pathway and the effect of D-pinitol. J. cachexia, sarcopenia muscle 15 (3), 934–948. 10.1002/jcsm.13459 38553831 PMC11154748

[B217] Zhenyukh O. Civantos E. Ruiz-Ortega M. Sánchez M. S. Vázquez C. Peiró C. (2017). High concentration of branched-chain amino acids promotes oxidative stress, inflammation and migration of human peripheral blood mononuclear cells *via* mTORC1 activation. Free Radic. Biol. and Med. 104, 165–177. 10.1016/j.freeradbiomed.2017.01.009 28089725

[B218] Zhu X. Li K. Liu G. Wu R. Zhang Y. Wang S. (2023). Microbial metabolite butyrate promotes anti-PD-1 antitumor efficacy by modulating T cell receptor signaling of cytotoxic CD8 T cell. Gut microbes 15 (2), 2249143. 10.1080/19490976.2023.2249143 37635362 PMC10464552

